# Selenoprotein DIO2 Is a Regulator of Mitochondrial Function, Morphology and UPRmt in Human Cardiomyocytes

**DOI:** 10.3390/ijms222111906

**Published:** 2021-11-02

**Authors:** Nils Bomer, Mario G. Pavez-Giani, Frederik E. Deiman, Annet N. Linders, Martijn F. Hoes, Christiane L.J. Baierl, Silke U. Oberdorf-Maass, Rudolf A. de Boer, Herman H.W. Silljé, Eugene Berezikov, Warner S. Simonides, B. Daan Westenbrink, Peter van der Meer

**Affiliations:** 1 Department of Cardiology, University Medical Centre Groningen, University of Groningen, P.O. Box 30.001, 9700 RB Groningen, The Netherlands; mario.pavez@med.uni-goettingen.de (M.G.P.-G.); f.e.deiman@umcg.nl (F.E.D.); a.n.linders@umcg.nl (A.N.L.); m.hoes@umcg.nl (M.F.H.); c.l.j.baierl@umcg.nl (C.L.J.B.); s.u.oberdorf@umcg.nl (S.U.O.-M.); r.a.de.boer@umcg.nl (R.A.d.B.); h.h.w.sillje@umcg.nl (H.H.W.S.); b.d.westenbrink@umcg.nl (B.D.W.); p.van.der.meer@umcg.nl (P.v.d.M.); 2 European Research Institute for the Biology of Ageing (ERIBA), University Medical Centre Groningen, University of Groningen, Antonius Deusinglaan 1, 9713 AV Groningen, The Netherlands; e.berezikov@umcg.nl; 3 Department of Physiology, Amsterdam University Medical Centre, Vrije Unversiteit Amsterdam, 1081 HV Amsterdam, The Netherlands; ws.simonides@amsterdamumc.nl

**Keywords:** heart failure, selenoproteins, DIO2, human cardiomyocytes, mtUPR, mitochondrial function, fetal-gene-program

## Abstract

Members of the fetal-gene-program may act as regulatory components to impede deleterious events occurring with cardiac remodeling, and constitute potential novel therapeutic heart failure (HF) targets. Mitochondrial energy derangements occur both during early fetal development and in patients with HF. Here we aim to elucidate the role of DIO2, a member of the fetal-gene-program, in pluripotent stem cell (PSC)-derived human cardiomyocytes and on mitochondrial dynamics and energetics, specifically. RNA sequencing and pathway enrichment analysis was performed on mouse cardiac tissue at different time points during development, adult age, and ischemia-induced HF. To determine the function of DIO2 in cardiomyocytes, a stable human hPSC-line with a DIO2 knockdown was made using a short harpin sequence. Firstly, we showed the selenoprotein, type II deiodinase (DIO2): the enzyme responsible for the tissue-specific conversion of inactive (T4) into active thyroid hormone (T3), to be a member of the fetal-gene-program. Secondly, silencing DIO2 resulted in an increased reactive oxygen species, impaired activation of the mitochondrial unfolded protein response, severely impaired mitochondrial respiration and reduced cellular viability. Microscopical 3D reconstruction of the mitochondrial network displayed substantial mitochondrial fragmentation. Summarizing, we identified DIO2 to be a member of the fetal-gene-program and as a key regulator of mitochondrial performance in human cardiomyocytes. Our results suggest a key position of human DIO2 as a regulator of mitochondrial function in human cardiomyocytes.

## 1. Introduction

The heart itself exhibits an extraordinary capacity to adapt to long-term changes when in need [1]. For example, postnatal growth, exercise, and pregnancy have the commonality that they all result in increased hemodynamic loading. To be able to counteract these increased pressures, the heart will respond with physiological cardiomyocyte hypertrophy [2,3,4]. These long-term adaptations were found, to some extent, recapitulated as the result of cardiac injury [3]. The reoccurrence of transcriptional processes specific for early development, has been depicted as “cardiac fetal reprogramming” and is characterized by the re-activation of fetal gene transcription profiles in the diseased myocardium [5]. This adaptation is suggested to act as a dynamic mechanism in demand, counteracting detrimental processes occurring in the diseased heart during cardiac remodeling, and therefore constitutes a pro-survival reaction. The increase of active thyroid hormone-converting enzymes has been related to this compensatory process. In particular, the deiodination of thyroid hormones T_3_ and T_4_ is an integral component of thyroid hormone homeostasis. Iodothyronine Deiodinases (DIO’s) participate in the hormone activity modulation, mediating the deiodination catalysis of T_3_ and T_4_ [6,7]. More specifically, Iodothyronine Deiodinase type II (DIO2) is a selenoprotein family member, expressed in multiple tissues, such as brown adipose tissue, pituitary gland, brain, cartilage, and the heart, amongst others. In the myocardium, DIO2 is primarily located in the endoplasmatic reticulum, where it converts intracellular thyroid hormone from its inactive (T_4_) into its active form (T_3_) and is responsible for two-thirds of tissue-specific T_3_ production mostly in the response to stress [8,9]. The heart is a principal target of thyroid hormone signaling and required in the neonatal and developing heart. Indeed, a cross-sectional study demonstrated that 18.5% of the patients with congenital hypothyroidism presented cardiac defects [10]. On the other hand, subtle changes in thyroid hormone signaling are implicated in pathological ventricular remodeling and the development of heart failure [10,11]. Mitochondrial dysfunction has been highlighted as a pathophysiological mechanism underlying reduced thyroid hormone signaling [8,11,12,13] and could be a potent target for therapy to improve cardiac function directly [14,15,16,17,18,19,20]. Abnormalities in mitochondrial function can result in reduced respiration, increased reactive oxygen species production, altered utilization of metabolic substrates, aberrant mitochondrial morphology, and impaired calcium homeostasis [14]. Some controversy exists concerning the function of DIO2 induction as a consequence of cardiac damage. Identification and a better understanding of key regulatory components on the crossroad of fetal reprogramming and mitochondrial dynamics, such as DIO2, may constitute potential novel therapeutic HF targets. Here, we show that DIO2 is part of the fetal-gene-program and reoccurs during HF. Silencing DIO2 expression in human cardiomyocytes severely affects several facets of mitochondrial dynamics.

## 2. Results

### 2.1. Identifying Genes and Pathways Involved in the Expression of the Cardiac Fetal-Gene-Program upon Heart Failure

**Figure 1:**
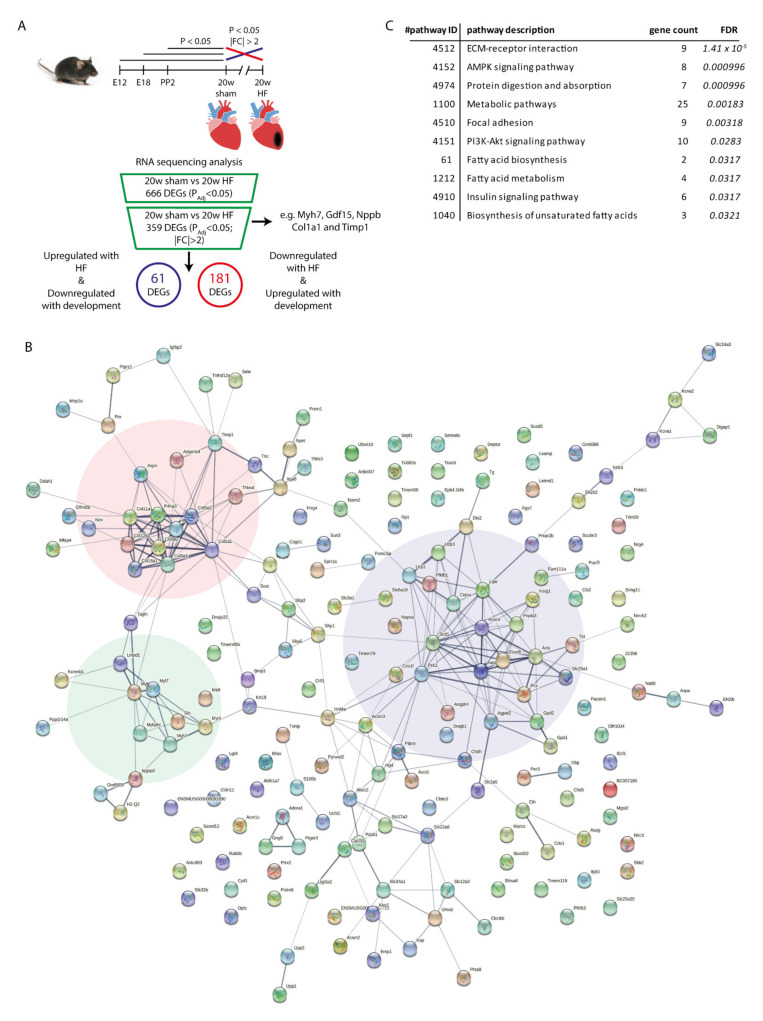
Overview of study strategy and graphical representation of the data. (**A**) Graphical representation of the study design and RNA sequencing analysis. The flowchart showing the identification of genes contributing to the recapitulation of the fetal gene program. For each time point, *n* = 3 animals were used. (**B**) Protein-protein interactions (STRING-db) for de 242 differentially expressed genes (DEGs) contributing to the recapitulation of the fetal gene program. Clusters within the protein-protein interactions were identified for metabolism (blue), ECM (red) and sarcomere structure (green). (**C**) Pathway enrichment analysis identified several KEGG pathways to be enriched in the dataset.

To identify the genes and pathways associated with the cardiac fetal-gene-program, we performed RNA-sequencing using RNA from mouse whole heart at embryonic day 12 (E12), left ventricular (LV) tissue at three different stages of cardiac development [E18, postpartum day 2 (PP2), and week 20 sham], and LV tissue of ischemia/reperfusion (IR)-induced HF (week 20-IR) (Figure 1A). To identify genes differentially expressed in HF, week 20 sham was compared with week 20 IR (Figure 1B). We identified a total of 360 differentially expressed genes (DEG) (FDR < 0.05 and |FC| > 2) in IR-induced HF (103 up-regulated genes and 257 down-regulated genes; Supplementary Table S3).

Genes involved in the activation of the cardiac fetal gene program were defined as genes that demonstrated an inverse regulation in HF as compared to that during early development. To identify these inversely up-regulated or down-regulated genes, we compared each of the developmental time points (embryonic day 12 (E12), day 18 (E18) and postpartum day 2 (PP2)) with the adult time-point (week 20 Sham) for the genes differentially expressed in HF. Genes showing differential expression (DEG; *P_FDR_* < 0.05) at one point during development inverse to the direction with HF were selected. We identified 61 DEGs inversely up-regulated with HF and 181 DEGs inversely down-regulated with HF compared to a development stage (Supplementary Table S4). Based on GO-term enrichment analysis, the DEGs up-regulated with HF were highly enriched for genes involved in biological processes: cell adhesion (GO:0007155; *P_FDR_* = 4.7 × 10^−6^) extracellular matrix organization (GO:0030198; *P_FDR_* = 2.99 × 10^−5^), collagen fibril organization (GO:0030199; *P_FDR_* = 6.76 × 10^−5^) and response to stress (GO:0006950; *P_FDR_* = 0.00361), including Col1a1, Col3a1, Postn, Wisp1 and Srfp2 (Table S3) and coding for proteins localized in the proteinaceous extracellular matrix (GO:0005578; *P_FDR_* = 6.26 × 10^−19^). DEGs down-regulated with HF were highly enriched for genes involved in biological processes: lipid metabolic process (GO:0006629; *P_FDR_* = 3.12 × 10^−6^), small molecule metabolic process (GO:0044281; *P_FDR_* = 3.12 × 10^−5^), and response to hormone (GO:0009725; *P_FDR_* = 8.44 × 10^−3^) including Acaca, Acly, Hnf4a, Ucp3 and Pck1 (Table S2) and coding for proteins localized in the mitochondrial envelope (GO:0005740; *P_FDR_* = 6.58 × 10^−3^).

Using the online tool STRING-db [21] to investigate protein-protein interactions (PPI), we found significant protein-protein interaction enrichment (*p* < 1.0 × 10^−16^; Figure 1B and Supplementary Figure S1). The KEGG (Kyoto Encyclopedia of Genes and Genomes) pathway enrichment analysis showed that the total of 242 DEGs were enriched for ECM-receptor interaction (pathway ID 04512; *P_FDR_* = 1.41 × 10^−5^) and metabolic pathways (pathway ID 01100; *P_FDR_* = 1.83 × 10^−3^), but also signaling pathways, such as AMP-activated protein kinase (AMPK) (*P_FDR_* = 9.96 × 10^−4^), PI3K-AKT (*P_FDR_* = 0.028) and insulin signaling (*P_FDR_* = 0.032) (Figure 1C). AMPK and PI3K-AKT signaling and insulin resistance in cardiomyocytes have in common that they are regulated by thyroid hormone signaling [4,22,23]. In addition, the T3 converting enzyme type II deiodinase (Dio2; *P_FDR_* = 0.022 and FC = 3.4) and the thyroid hormone precursor thyroglobulin (Tg; *P_FDR_* = 0.03 and FC = 2.1), the T_3_ converting enzyme and thyroid hormone precursor, respectively, were both up-regulated during development and in HF relative to week 20 sham mice. The up-regulation of Dio2 was found to be a generic event in heart failure of different etiologies in animal models (TAC, MI, I/R, Angiotensin II (ANGII) and hypertensive REN2-transgenic rats), in human in vitro models (Hypoxia/Oxygenation and ANGII treatment) and in human (ischemic and dilated) cardiomyopathy (Supplementary Methods and Figure S2).

Assessing *DIO2* expression during early human PSC-derived cardiomyocytes (hPSC-CMs) differentiation (day 0–12) also showed *DIO2* expression to sharply peak at day 6 of differentiation (initiation of spontaneous contractions) and rapidly decrease until fully differentiated (Supplementary Figure S2I).

### 2.2. Silencing DIO2 in hPSC-CMs Has No Effect on Gross Morphology

**Figure 2:**
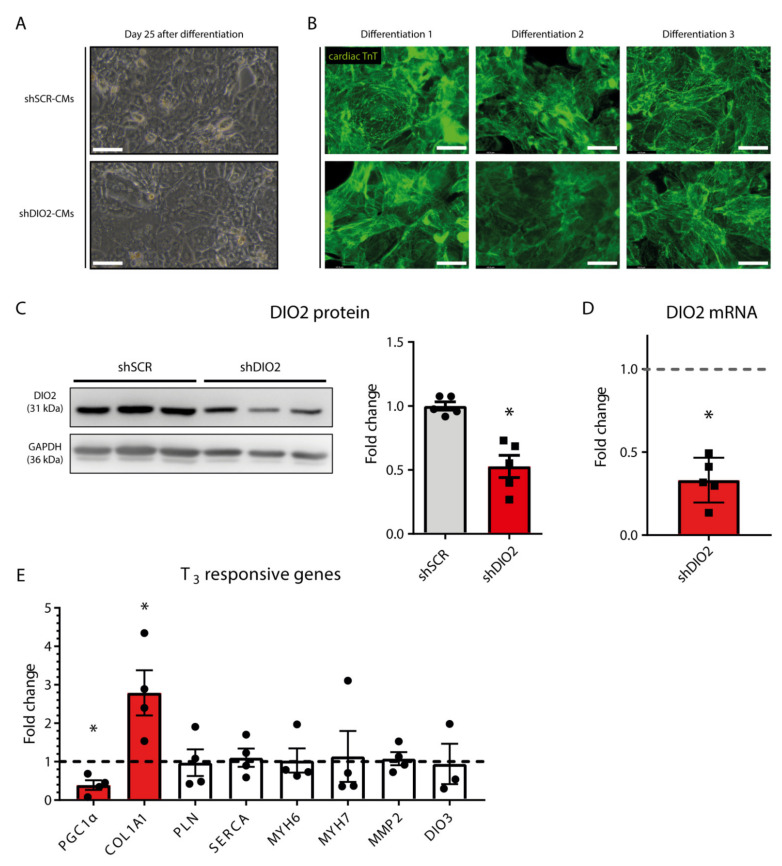
Gross cellular phenotyping of *DIO2* knockdown in hPSC-CMs. (**A**) Representative bright field images of shSCR- and shDIO2-CMs, 25 days after the start of differentiation showing no gross morphological differences (scale bar represents 100 µm). (**B**) Immunofluorescent staining for DAPI and cTnT in *n* = 3 independent differentiations of shSCR- and shDIO2-CMs (scale bar represents 43.9 µm). (**C**) Western blot results showing expression of DIO2 protein (*n* = 6 independent samples; corrected for GAPDH) and (**D**) RT-qPCR results showing expression of *DIO2* mRNA (*n* = 5 independent samples; corrected for 36B4). (**E**) RT-qPCR derived expression profiles of known T_3_ responsive genes (*PGC1α, COL1A1, PLN, SERCA, MYH6, MYH7, MMP2* and *DIO3*; *n* = 4 independent samples; corrected for 36B4). The dashed lines represent the relative expression levels of shSCR-CMs; * *p* < 0.05; Student unpaired *t*-test; values are compared to the mean shSCR-CM level. Data shown are expressed as means ± standard error of the mean (SEM).

Short hairpin RNA-mediated specific silencing of *DIO2* (shDIO2) did not affect the capacity of hPSCs to differentiate into cardiomyocytes (spontaneous beating between day 6–8), nor did it affect gross cellular morphology or expression of the cardiac marker α-actinin, nor its sarcomeric organization (Figure 2A,B), verifing unaffected differentiation towards cardiomyocytes. Assessing the effectiveness on mRNA levels of silenced *DIO2* in hPSC-CMs (shDIO2-CMs), showed an ~80% decrease in the shDIO2-CMs as compared to the control cells transduced with a short hairpin against a scrambled sequence (shSCR-CMs; Figure 2C,D). Western blot analysis revealed an ~50% decrease in the DIO2 protein in shDIO2-CMs reflecting the effective knockdown of *DIO2* (Figure 2C). Furthermore, *DIO2* knockdown led to the decreased mRNA expression of the established T_3_ responsive gene Peroxisome proliferator-activated receptor gamma coactivator 1-α (*PGC1α)* (FC = 0.39; *p* < 0.05). Furthermore, the increased expression of Collagen 1A1 (*COL1A1*) (FC = 2.8; *p* < 0.05) was found. However, no effect was observed for other known T_3_ responsive genes Myosin heavy chain-α (*MYH6)*, Myosin heavy chain-β (*MYH7)*, Phospholamban (*PLN)*, Sarco/endoplasmic reticulum Ca^2+^-ATPase (*SERCA)*, Metrix Metallopeptidase 2 (*MMP2)* or type III deiodinase *DIO3* (Figure 2E).

### 2.3. DIO2 Knockdown Impairs mtUPR and Transcriptional Landscape of Metabolic Genes, Resulting in Increased ROS and Decreased Viability

**Figure 3:**
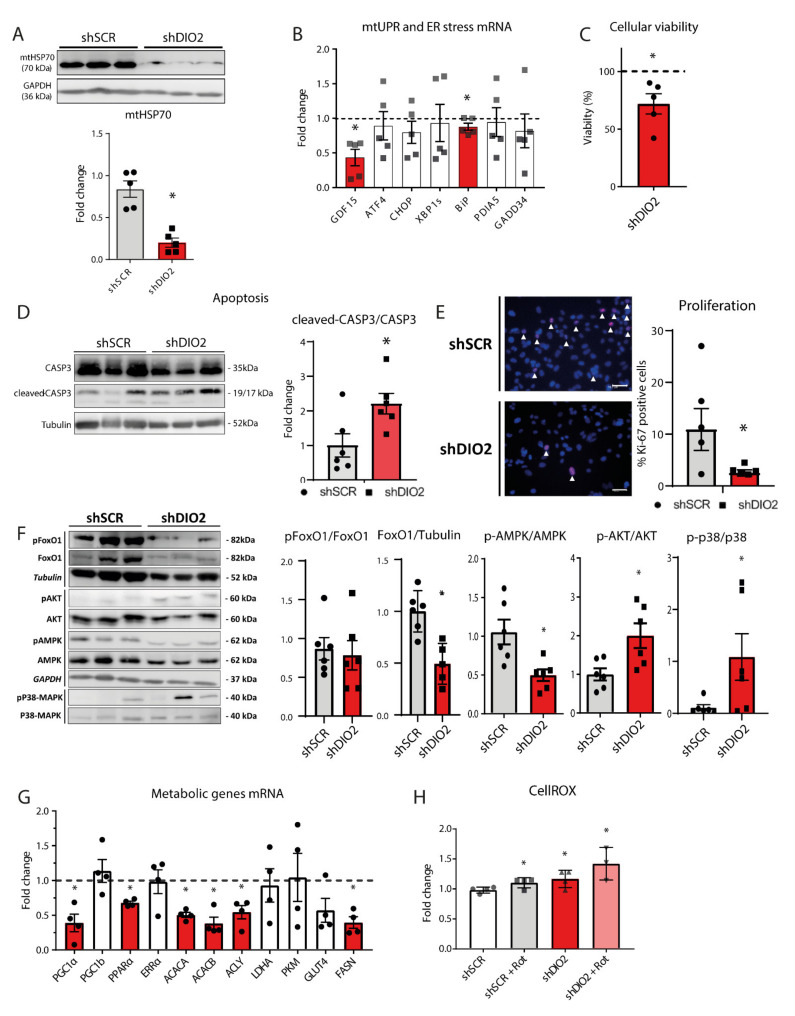
*DIO2* knockdown results in impaired mtUPR activation, increased ROS and decreased viability and an impaired metabolic transcriptional landscape. (**A**) Western blot results for mtHSP70 protein levels (*n* = 6 independent samples; corrected for GAPDH). (**B**) RT-qPCR derived expression profiles of genes involved in mitochondrial UPR and ER stress and ER stress-induced apoptosis (*GDF15, ATF4, CHOP, XBP1s, BiP, PDIA5* and *GADD34; n* = 5 independent samples; corrected for 36B4). (**C**) Baseline assessment of cellular viability (*n* = 5 independent samples). (**D**) Western blot analysis results showing levels of total Caspase 3 and cleaved–Caspase 3 and the calculated ratio (*n* = 6 independent samples). (**E**) Immunofluorescence staining of Ki-67 indicating proliferative activity in percentage positive cells (scale bar represents 50 µm; *n* = 5 independent samples). (**F**) Western Blot analysis results showing protein levels of (phosphorylated)-FoxO1/FoxO1, FoxO1/Tubulin, p-AMPK/AMPK, p-AKT/AKT and p-p38 MAPK/p38-MAPK (*n* = 6 independent samples). (**G**) RT-qPCR derived expression profiles of cardiac metabolic genes (*PGC1α, PGC1β, PPARα, ERRα, ACACA, ACACB, ACLY, LDHA, PKM, GLUT4 and FASN; n* = 4 independent samples; corrected for 36B4). (**H**) Cellular ROS levels in shSCR- and shDIO2-CMs under control conditions and with the addition of rotenone to induce oxidative stress (*n* = 4 independent samples). The dashed lines represent the relative expression levels of shSCR-CMs; * *p* < 0.05; Student unpaired *t*-test; values are compared to the mean shSCR-CM level. Data shown are expressed as means ± standard error of the mean (SEM).

Strikingly, in shDIO2-CMs, protein levels of mitochondrial Heat Shock Protein 70 (mtHSP70) were found drastically reduced (~4-fold decrease; Figure 3A). Additional RT-qPCR results showed a similar range of reduction in mRNA expression for mitochondrial stress marker Growth/Differentiation Factor 15 (GDF15). No effects of the DIO2 knockdown were observed on Activating Transcriptional Factor 4 (ATF4) or genes involved in Endoplasmic Reticulum (ER) stress (DNA damage-inducible transcript 3 (CHOP), X-box binding protein 1 (XBP1s), and Protein disulfide isomerase family A member 5 (PDIA5)), and ER stress induced apoptosis (GADD34; Figure 3B). Only a small reduction of Binding immunoglobulin protein (BiP) expression was observed. Staining for antigen Ki-67 showed a reduced proliferative capacity of the shDIO2-CMs (10.9% vs. 2.5%; Figure 3E). Furthermore, apoptosis was assessed by determining the ratio between cleaved and uncleaved Caspase 3 (CASP3). Apoptosis was found significantly increased in shDIO2-CMs (Figure 3D). This was in line with the observation of a 30% (*p* < 0.05) reduction in the cellular viability in the shDIO2-CMs (Figure 3C).

Next, a clear reduction in Forkhead box protein O1 (FoxO1) protein levels (Figure 3F) and phosphorylated AMPK (Thr-172; Figure 3F) was observed. These findings were aligned with the transcriptional metabolic profile of shDIO2-CMs, with a significant overall reduction of genes involved in mitochondrial biogenesis and fatty acid metabolism, such as *PGC1α,* Peroxisome proliferator activated receptor α *(PPARa),* Acetyl-CoA Carboxylase α *(ACACA),* and β *(ACACB)*, ATP citrate lyase (*ACLY)*, and Fatty acid synthase (*FASN)* (Figure 3G). Cellular reactive oxygen species (ROS) levels were found to be increased in shDIO2-CMs (17%; *p* < 0.05) as compared to shSCR-CMs (Figure 3H), which is consistent with the increase of Glutathione peroxidase 1(*GPX1)* mRNA expression (FC = 1.6; *p* < 0.05). Incubating the cells with rotenone resulted in a stronger increase in cellular ROS (42%; *p* < 0.05). Elevated ROS production and the observed augmentation of p38-MAPK phosphorylation in shDIO2-CMs (Figure 3F) are tightly related. Further Western blot analyses showed that the AKT signaling pathway was not negatively affected by DIO2 silencing, with an observed 2-fold increase of AKT phosphorylation (Figure 3F).

### 2.4. DIO2 Knockdown Impairs Mitochondrial Function, Biogenesis and Morphology

**Figure 4:**
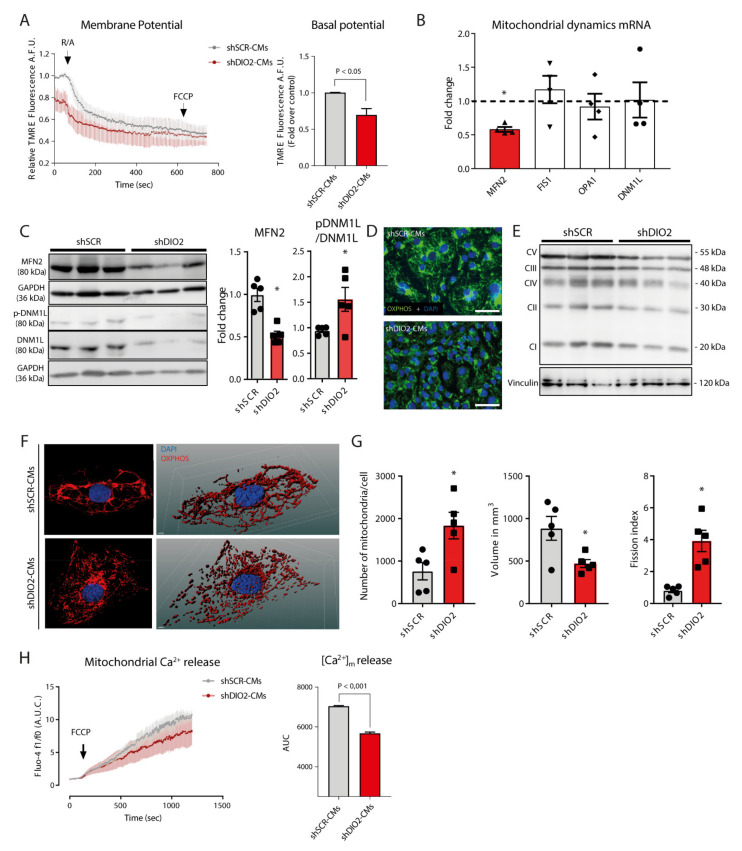
Mitochondrial profiling of shDIO2-cardiomyocytes. (**A**) Relative TMRE fluorescence indicating membrane potential and basal potential difference (*n* = 3 independent samples). (**B**) RT-qPCR derived expression profiles of genes involved in mitochondrial dynamics (*MFN2, FIS1, OPA1* and *DNM1L;* (*n* = 4 independent samples) corrected for 36B4). The dashed line represents the relative expression levels of shSCR-CMs; * *p* < 0.05. (**C**) Western blot results of MFN2 ((*n* = 6 independent samples) corrected for GAPDH) and (phosphorylated) DNM1L (corrected for non-phosphorylated DNM1L) protein levels (*n* = 6 independent samples) (**D**) Representative immunofluorescent staining for DAPI and OXPHOS in shSCR- and shDIO2-CMs (scale bar represents 50 µm). (**E**) Western blot results of OXPHOS complex protein levels (*n* = 6 independent samples; corrected for vinculin). (**F**) Representative mitochondrial network images of immunofluorescent staining for DAPI and OXPHOS and 3D convolution of shSCR- and shDIO2-CMs. (**G**) Relative mitochondrial volume/number and fission index (*n* = 5 independent samples). (**H**) Fluo-4 fluorescence levels indicating mitochondrial calcium release as the result of FCCP (*n* = 3 independent samples); * *p* < 0.05; Student unpaired *t*-test; values are compared to the mean shSCR-CM level. Data shown are expressed as means ± standard error of the mean (SEM).

Estimation of the mitochondrial membrane potential and its reduction upon blocking the electron transport chain showed significant lower membrane potential in shDIO2-CMs at baseline and over time (Figure 4A). Reduced mRNA expression of *PGC1α* and Mitofusin 2 (*MFN2*) was observed in shDIO2-CM, but not for *PGC-1β*, Mitochondrial fission 1 (*FIS1)*, Optic atrophy protein 1 (*OPA1*) or Dynamin 1 like (*DMN1L*) (Figure 4B). Western blot however showed increased DMN1L phosphorylation and reduced MFN2 protein expression levels in shDIO2-CMs (Figure 4C). No change in the Translocase of outer membrane subunit 20 (TOM20) protein levels was observed (Figure S2). Additionally, immunofluorescence staining of the inner mitochondrial membrane (Oxidative phosphorylation (OXPHOS) complexes) demonstrated a lower abundance of mitochondrial complexes in shDIO2-CMs (Figure 4D), which could be confirmed on Western blot (Figure 4E). 3D reconstruction of the mitochondrial network using z-stacks of the inner membrane staining (Figure 4F) showed an aberrant mitochondrial structure in shDIO2-CMs as reflected by significantly increased mitochondrial number/volume and decreased mitochondrial volume per cell resulting in an increased fission index (Figure 4G). Assessing mitochondrial calcium release as the consequence of Carbonyl cyanide-p-trifluoromethoxyphenylhydrazone (FCCP (20 µM)) induction revealed significantly reduced mitochondrial Ca^2+^ content in shDIO2-CMs (Figure 4H).

**Figure 5:**
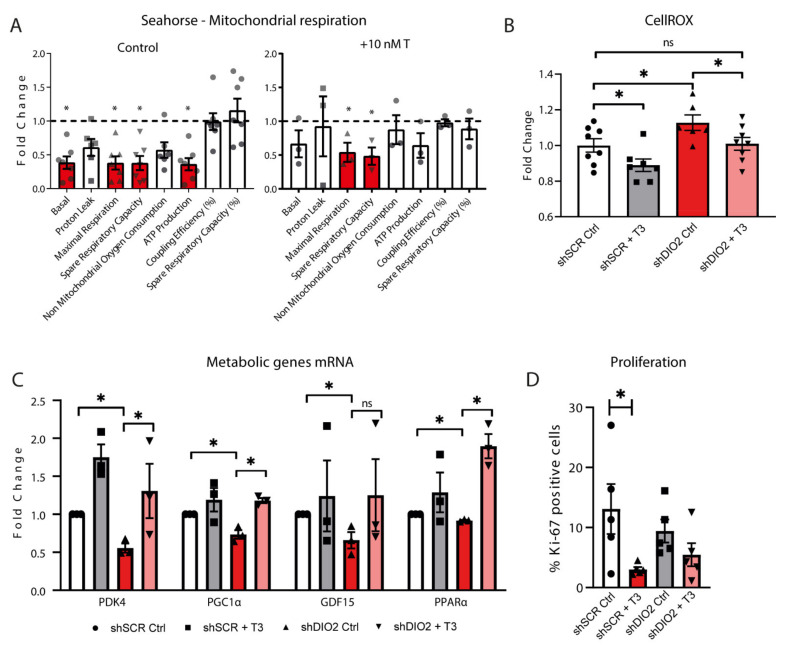
Rescue experiments using T_3_ in shDIO2-cardiomyocytes. (**A**) Effects of DIO2 knockdown (*n* = 7 independent samples) and T_3_ (10 nM) rescue (*n* = 3 independent samples) in shDIO2-CMs on basal respiration, proton leak, maximal respiration, respiratory reserve, non-mitochondrial oxygen consumption, ATP-linked respiration, coupling efficiency (%) and spare respiratory capacity (%). The dashed lines represent the relative expression levels of shSCR-CMs; * *p* < 0.05; Student unpaired *t*-test; values are compared to the mean shSCR-CM level. (**B**) Cellular ROS levels in shSCR- and shDIO2-CMs under control conditions and with the addition of T_3_ (10 nM) as a rescue experiment (*n* = 8 independent samples. (**C**) RT-qPCR derived expression profiles of cardiac metabolic genes (*PDK4*, *PGC1α, GDF15,* and *PPARα; n* = 4 independent samples; corrected for *36B4*) in rescue experiments with and without the addition of T_3_ (10 nM); ● = shSCR Ctrl; ■ = shSCR + T_3_; ▲ = shDIO2 Ctrl; ▼ = shDIO2 + T_3_. (**D**) Immunofluorescence staining of Ki-67 indicating proliferative activity in percentage positive cells ((*n* = 5 independent samples). * *p* < 0.05; Student unpaired *t*-test; values are compared to the mean shSCR-CM level. Data shown are expressed as means ± standard error of the mean (SEM).

Knockdown of DIO2 resulted in severely impaired mitochondrial respiration (Figure 5A). shDIO2-CMs showed a more than 2-fold reduction (*p* < 0.001) of basal oxygen consumption rate (OCR) compared to control shSCR-CMs (Figure 5A). Oligomycin A inhibited ATP synthase-linked respiration resulted in a significant reduction of ATP synthase-linked respiration (*p* < 0.001) (Figure 5A). The protonosphore FCCP disrupts mitochondrial membrane potential and promotes its maximal capacity, resulting in a more than 2-fold lower maximal capacity compared to the control condition (*p* < 0.005) (Figure 5A). The respiration reserve capacity, an estimate of the potential bioenergetic reserve, was unchanged in shDIO2-CMs (Figure 5A).

### 2.5. Mitochondrial Respiration, ROS Levels and Metabolic mRNA Profile in shDIO2-CMs Could Be Rescued by T3

A rescue experiment in which the shDIO2-CMs were treated for 7 days with T_3_ (10 nM) prior to seahorse measurement showed recovery of the mitochondrial basal respiration and ATP-linked respiration (Figure 5A; Supplementary Figure S4). Maximal respiration and spare respiratory capacity were still impaired (~50% reduction; *p* < 0.05) as compared to the shSCR-CMs. Cellular ROS levels were found to be increased in shDIO2-CMs as compared to shSCR-CMs (Figure 5B). Treating the shDIO2-CMs with T_3_ for 7 days resulted in a reduction of ROS, back to levels observed in shSCR-CMs under control conditions (Figure 5B). Furthermore, mRNA expression levels of Pyruvate dehydrogenase 4 (*PDK4)*, *PPARa*, *PGC1a* and *GDF15* were shown to be reversed to shSCR-CMs levels upon treatment with T_3_ (Figure 5C). However, expression levels of *p21*, *COL1A1* and *DIO2* were found to be independent of T_3_. Of note, Ki-67 staining showed that the reduction in proliferative capacity in shDIO2-CMs could not be completely reversed by T_3_ treatment (Figure 5D).

## 3. Discussion

We recognized *DIO2* as a member of the fetal-gene-program and identified it to be a regulator of mitochondrial function, morphology and UPR^mt^ in human cardiomyocytes. DIO2 induction showed to be a generic reaction in the myocardium resulting from a broad variety of HF etiologies in human and murine subjects. Silencing *DIO2* expression in human cardiomyocytes proofed to have severe detrimental effects, especially on reduced viability, increased ROS, severely impaired mitochondrial respiration, morphology and impaired UPR^mt^. 3D reconstruction of the cardiomyocyte’s mitochondria revealed a severely fragmented morphology. Adding T_3_ to the shDIO2-CMs for the greater part rescued mitochondrial respiratory functions, ROS levels and gene expression. These results suggest that local hypothyroid condition, resulting from impaired DIO2 availability for the conversion of T_4_ into T_3_, may be contributing to the severely impaired mitochondrial function, bioenergetic capacity and cardiomyocyte viability.

The UPR^mt^ is a transcriptional response that is activated by the accumulation of misfolded proteins in the mitochondria [24] and promotes cell survival and preserves mitochondrial function. The accumulation of misfolded proteins impairs mitochondrial protein import efficiency and results in subsequent mitochondrial dysfunction (OXPHOS disruption, ROS overload and mito/nuclear protein imbalance) [24]. Reducing DIO2 levels severely impairs UPR^mt^ activation, as depicted by the steep decrease of mtHSP70 and *GDF15* expression levels. The suggested hypothyroid-condition as the result of impaired DIO2 expression reduces mtHSP70 expression [25], coincident with decreased protein import rates in the mitochondria [26,27]. Moreover, mtHSP70 may play a role in the control of cell proliferation [28], which was found reflected in the reduced percentage of Ki-67 positive nuclei in shDIO2-CMs (Figure 3E).

Furthermore, silencing *DIO2* in human cardiomyocytes resulted in impaired regulation of known genomic (*PGC1α* and *COL1A1*), as well as nongenomic targets (p38-MAPK, AMPK and FoxO1) of tissue thyroid hormone signaling [8]. Also, we observed an overall transcriptional down-regulation in the mRNA of a cardiac metabolic-panel which was in line with FoxO1 protein level reduction and subsequent decreased *PDK4* and *PPARa* gene expression [29]. The changes observed seemed to be independent of (*Thr473)* AKT phosphorylation, which was found increased. It could be suggested that silencing *DIO2* does not influence AKT activation directly, but triggers a natural stress-response to compensate the effects on mitochondrial dynamics and ROS levels [30].

Mitochondrial morphology and calcium load were shown to be severely impaired in the shDIO2-CMs. Active intracellular T_3_ is known to positively regulate de novo biogenesis of the mitochondria and is a stimulator of the pAMPK-PGC1α pathway [31,32], which was shown down-regulated in the shDIO2-CMs. Subsequently, the increase of DMN1L activation and the reduction of MFN2 expression suggest a balance shift towards active fission of mitochondria. 3D reconstruction of the mitochondrial network indeed showed an increased number of mitochondria per volume (fission index). The observed impairment in the mitochondrial calcium load may be the result of reduced mitochondrial potential [33] as the consequence of defective UPR^mt^ activation, as observed in shDIO2-CMs [34]. In the shDIO2-CMs, lower basal oxygen respiration of the electron transport chain was accompanied by lower membrane potential and lower mitochondrial calcium content, reflecting the proposed concept of impaired homeostasis of the mitochondria [34].

Whether the effects we observed on the mitochondrial dynamics are the result of local hypothyroidism due to reduced deiodinase activity, were assessed by culturing shDIO2-CMs in the presence of T_3_ (10nM)_._ After 7 days the reduction of basal and ATP-linked respiration due to *DIO2* silencing were compensated. Furthermore, effects on mRNA expression of genes related to mitochondrial biogenesis and substrate oxidation (*PGC-1α, GDF15, PDK4* and *PPARa*) were reverted by T_3_. In addition, cellular ROS levels returned to control levels after T_3_ treatment. This implies that the effects of impaired *DIO2* on mitochondrial function are through reduced tissue-specific T_3_ conversion. However, since these observations were indirect, we cannot exclude that the mechanism of the abnormalities observed are beyond T_3_ activity.

Reoccurrence of the fetal-gene-program, because of cardiac stress, has been proposed as a compensatory mechanism to handle the reduced hemodynamics as the result of cardiac muscle damage or overload. The increase of the T_3_-generating protein DIO2 is suggested to be an aspect of this compensatory process. However, some controversy exists concerning the function of DIO2 induction as a result of cardiac damage. A conventional view is that hypertrophic growth of the heart is an adapting response to increased hemodynamic loading. This compensatory mechanism acts to reduce wall stress and prevents the reduction of the cardiac contractile function. It was suggested that the overexpression of *DIO2*, and subsequent increased intracellular T_3_ availability, is a crucial step in the recovery processes after e.g., myocardial infarction, pressure overload, or doxorubicin-induced cardiotoxicity in mouse models [35,36,37,38,39,40,41]. However, very recently DIO2 up-regulation was shown to be involved in murine maladaptive hypertrophy under the transcriptional control of FoxO1 overexpression [42]. Our results assume that the DIO2-FoxO1 signaling axis is not one-way traffic, as was reviewed by van der Vos and Coffer [43]. They showed that FoxO1 could be associated with a variety of unrelated transcription factors, regulating activation or repression of diverse target genes. The complement of transcription factors expressed in a particular cell type is thus critical in determining the functional end point of FoxO1 activity. One of the unrelated transcription factors that regulates the FoxO1 target gene expression is the thyroid hormone receptor [43], showing that the function and activity of FoxO1 is dependent on the active intracellular conversion of thyroid hormone, T_3_. Together with the impaired activation of AMPK, the increased activation of p38-MAPK, and increased ROS levels, this suggests that cardiomyocytes deprived from DIO2 are unable to commence stress-induced physiological cardiac hypertrophy [44], despite activation of the AKT pathway. Consequently, both sides of the spectrum are suggested to be detrimental for proper functioning of the heart. On one side, the inability of increasing DIO2 expression with stress hampers necessary mitochondrial dynamics, by which the myocardium becomes energy deprived. On the other side, the vast overexpression of DIO2 over a longer period induces hypertrophic overcompensation, by which this becomes pathologic.

There is no genetic variation known for DIO2 impairment that is associated with the onset of HF, CAD, stroke, or associated mortality in large GWA studies. However, there are causes known in patients that could cause the inadequate expression of *DIO2* expression and/or function as the result of cardiac stress. Low plasma concentrations of several micronutrients have been associated with a reduced quality of life and adverse outcomes in HF [45,46,47,48,49]. Most patients with HF consume less than the recommended daily amount of several micronutrients [50,51], with vitamin D (97% of patients) and selenium (95%) intake being most often inadequate [52], and up to 50% are deficient in one or more micronutrients in cross-sectional studies [50,52,53]. Selenium is a component of selenocysteine [54], an amino acid that is required for the formation of selenoproteins [9,55,56], including glutathione peroxidases (GPXs), thioredoxin reductases (TXNRDs), and iodothyronine deiodinases (DIOs). The occurrence of selenium deficiency, which was found in almost 25% of HF patients in a large European cohort (*n* = 2382) and was associated with reduced exercise capacity and a substantially higher mortality in a prospective cohort study [49,57]. Selenium deficiency causes the cell to be unable to synthesize selenoproteins. Health effects resulting from low Se intake is thought to be caused by insufficiency of specific selenoproteins. Dietary Se intake was shown to be essential for activity of the selenoprotein *DIO2* [9,58]. Se deficiency reduced the enzymatic activity responsible for the transformation of T_4_ to T_3_, which has implications for the expression of key cardiac genes in development and in heart failure [8].

Our results, in addition to the conflicting data from animal studies, highlight the need to elucidate the temporal expression patterns of DIO2 in the stressed myocardium. It might be hypothesized that the induction of DIO2 could be beneficial in the first days after cardiac injury, while induction over a longer period could lead to overcompensation and consequently, pathological hypertrophy. Considering the direct clinical implications (selenium-deficiency), regulation of DIO2 expression should be tightly balanced when addressing potential therapeutic properties for future treatment options for heart failure.

## 4. Materials and Methods

### 4.1. Animal Discovery Model, RNA-Seq Library Preparation and Sequencing, and Analysis

All animal protocols were approved by the Animal Ethical Committee of the University of Groningen (permit number DEC6002AA; 26-01-2016). All procedures must conform to the guidelines from Directive 2010/63/EU of the European Parliament on the protection of animals used for scientific purposes or the NIH Guide for the Care and Use of Laboratory Animals. We used an existing dataset generated in our lab as previously described [59]. In short, we used a total of three stages of murine cardiac development (E12, E18 and PP2) from this study. For E12, the whole heart was removed for each embryo. At E18, the left ventricle was excised from the embryos. The left ventricles of PP2 pups were removed after decapitation. Also, the left ventricles from 20-week-old mice were included that had undergone ischemia/reperfusion (IR) injury or sham treatment. For each time point, *n* = 3 samples were used. Libraries were generated by strictly following NEXTflex Illumina RNA-Seq Library Prep version 2 kit recommendations (Bioo Scientific (Austin, TX, USA)). The libraries were sequenced on the Illumina HiSeq 2500 machine (San Diego, CA, USA) in the RapidRun mode.

Reads were mapped to the mouse genome assembly GRCm38 using STAR aligner version 2.5.2b [60]. Gene counts were derived from the alignment files using the HTSeq-count program version 0.6.1p1 from HTSeq package [61] and Ensembl gene annotations. Differential gene expression between pairs of conditions was calculated using edgeR package [62] with batch effects correction and upper quartile normalization. Genes with FDR of ≤0.05 were considered as statistically significant.

### 4.2. Lentiviral Production and Transduction of hPSC-CMs

HEK-293T cells were cultured at 37 °C and 5% CO_2_ in Dulbecco modified Eagle medium (DMEM; 41965-039, Thermo Fisher Scientific (Waltham, MA, USA)) supplemented with 10% fetal calf serum (FCS; F7524, Sigma-Aldrich (St. Louis, MO, USA)). HEK-293T were transfected with Fugene HD (E2311, Promega (Madison, WI, USA)) and a mix of pCMV ∆8.91-transfer plasmid, VSV-G-packaging plasmid and pLKO.1-plasmid expressing short hairpin RNA (shRNA) against DIO2 or a non-mammalian scrambled sequence at a ratio of 5:2:6. Media were replaced with a fresh Essential 8 (E8) medium after 24 h. An E8 medium containing viral particles was harvested and filtered with a 0.45 nm Nalgene filter after 48 h. A clean viral supernatant was used directly or was snap frozen for extended storage. pLKO.1.shDIO2 (TRCN0000084067, Sigma-Aldrich) and pLKO.1.shSCR (SHC002, Sigma-Aldrich (St. Louis, MO, USA)) were purchased from Sigma MISSION RNAi. HUES9 cells (Harvard Stem Cell Institute (Cambridge, MA, USA); hPSCs) were plated as described before and infected with an aliquot of clean viral supernatant supplemented with an additional E8 supplement in the presence of 8 µg/mL of polybrene for 24 h. After the first 24 h, a normal E8 medium is then changed every day. Subsequently, 1 μg/mL of puromycin (A1113803; Thermo Fisher Scientific) was added to the culturing medium to select for transduced hPSCs.

### 4.3. Cell Culture, Cardiomyocyte Differentiation and Cardiac Stress Models

The hPSCs were maintained in an Essential 8 medium (A1517001; Thermo Fisher Scientific (Waltham, MA, USA)) on a Geltrex^®®^-coated surface (A1413301; Thermo Fisher Scientific (Waltham, MA, USA)) under controlled conditions with 37 °C, 5% CO_2_ and 100% atmospheric humidity, and the medium was refreshed daily. Differentiation to cardiomyocytes was achieved as described previously [49,63,64,65]. Briefly, hPS cells were dissociated with 1× TrypLE (12604-021; Thermo Fisher Scientific (Waltham, MA, USA)) for 4 min and plated as single cells in an Essential 8 medium containing 5 μM Y26732 (S1049, Selleck Chemicals (Houston, TX, USA)), The Essential 8 medium (without Y26732) was refreshed daily. Once cultures reached 80% confluency, the cells were washed with PBS and differentiation was initiated (day 0) by culturing cells in an RPMI1640 medium (21875-034, Thermo Fisher Scientific (Waltham, MA, USA)) supplemented with 1× B27 minus insulin (Thermo Fisher Scientific (Waltham, MA, USA)) and 6 μM CHIR99021 (13122, Cayman Chemical (Ann Arbor, MI, USA)). At day 2, the cells were washed with PBS and the medium was refreshed with RPMI1640 supplemented with a 1× B27 minus insulin and 2 μM Wnt-C59 (5148, Tocris Bioscience (Bristol, UK)). From day 4, the medium was changed to a CDM3 medium as described by Burridge et al. [65] and was refreshed every other day as a maintenance medium. This resulted in cultures with >90% spontaneously contracting cardiomyocytes at days 8–10. To further enrich these cultures, the cells were dissociated and replated at day 12 in a glucose-free RPMI1640-based (11879, Thermo Fisher Scientific (Waltham, MA, USA)) CDM3 medium supplemented with 5 mM sodium dl-lactate (CDM3L; L4263, Sigma-Aldrich (St. Louis, MO, USA)) for 4–6 days [65]. To increase the survival of cardiomyocytes after the dissociation, a 2% KnockOut Serum Replacement (KOSR, Thermo Fisher Scientific (Waltham, MA, USA)) was added. The CDM3L medium was refreshed every other day. This resulted in >99% pure spontaneously beating cardiomyocytes. By itself, lactate treatment (CDM3L) was shown to promote the expression of a fetal-like program [66]. Therefore, after lactate selection (days 16–18) the cardiomyocytes are cultured an additional 12–14 days in a CDM3 medium to revert the effects of lactate selection. Experiments were typically started at day 30 from the start of differentiation, after once more dissociating the cells to the experimental set-up in CDM3 medium which was refreshed every other day. To induce oxidative stress in the hPSC-CMs, 3 µM Rotenon (R8875, Sigma-Aldrich (St. Louis, MO, USA)) was added to the culture medium. This compound suppresses complex I of the mitochondrial respiratory chain and inhibits NADH oxidation, thereby causing the overproduction of reactive oxygen species. For rescue experiments, fully differentiated hPSC-CMs were cultured with 10 nM T_3_ (R8875, Sigma-Aldrich (St. Louis, MO, USA)) added to the CDM3 culture medium for 7 days. The medium was refreshed every other day.

### 4.4. Western Blot and RT-qPCR

Protein was isolated in a Radioimmunoprecipitation assay (RIPA) buffer supplemented with 1% phosphatase inhibitor cocktail 3 (p0044, Sigma-Aldrich (St. Louis, MO, USA)), 1× cOmplete protease inhibitor cocktail (11873580001, Roche (Basel, Switzerland)), and 15 mM sodium orthovanadate (S6508, Sigma-Aldrich (St. Louis, MO, USA)). Protein concentration was determined with the DC protein assay kit. Equal amounts of protein were separated by SDS-PAGE and proteins were transferred to PVDF membrane. Antibodies for detection of specific proteins are listed in Supplementary Table S1. Signals were detected visualized with Enhanced Chemiluminescence (ECL; NEL120001EA, PerkinElmer (Waltham, MA, USA)) and densitometry has been analyzed with ImageQuant LAS 4000 (GE Healthcare (Chicago, IL, USA)). Protein signals were normalized to respective GAPDH levels.

To analyze gene expression, total RNA was isolated using TRI reagent according to the provided protocol (T9424, Sigma (St. Louis, MO, USA)). RNA concentrations have been determined with a Nanodrop 2000 (Thermo Scientific (Waltham, MA, USA)), and cDNA was synthesized using the QuantiTect Reverse Transcription kit (205313, Qiagen (Hilden, Germany)). Gene expression analysis was performed by qRT-PCR using IQ SYBR Green (170-8885, BioRad (Hercules, CA, USA)). The samples were normalized to the reference gene ribosomal protein lateral stalk subunit P0 (*36B4*). One separate differentiation was depicted as one N and to handle with the high variability in differentiations of primary cardiomyocytes, we treated every internal control within each experiment as self-contained. Relative expression differences were calculated by means of the ∆∆Ct-method for each experiment. A dotted line was used to visualize the summary of controls. The primers used can be found in Supplementary Table S2.

### 4.5. Immunofluorescence

The hPSC-CMs on coverslips were washed twice with cold PBS, and fixed with 4% paraformaldehyde on ice for 10 min. Fixed hPSC-CMs were washed 3 times with PBS, followed by permeabilization with PBS + 0.3% Triton-X100 (T9284, Sigma-Aldrich (St. Louis, MO, USA)) on ice for 5 min. Samples were blocked for 1 h at room temperature with PBS/Tween (0.1%; P1379, Sigma-Aldrich (St. Louis, MO, USA)) containing 3% BSA (11930, Serva (Heidelberg, Germany)) and 2% donkey serum (D9663, Sigma (St. Louis, MO, USA)). The hPSC-CMs were subsequently incubated with polyclonal anti-cardiac troponin T IgG (1:100; ab45932, Abcam(Cambridge, UK)), Total OXPHOS Rodent WB Antibody Cocktail (1:400; ab110413, Abcam (Cambridge, UK)) and monoclonal anti-Ki-67 (1:200; MA5-14520, Thermo Fisher Scientific (Waltham, MA, USA)) diluted in the blocking mix for 1 h. After washing, the cells were incubated with Alexa Fluor 488 donkey-anti-mouse IgG (1:1000; A21202, Thermo Fisher Scientific (Waltham, MA, USA)) or Alexa Fluor 555 donkey-anti-rabbit IgG (1:1000; A31572, Thermo Fisher Scientific (Waltham, MA, USA)). Coverslips were mounted with Vectashield mounting medium containing DAPI (H-1200, Vector labs (Burlingame, CA, USA)) and images were obtained with a Leica AF-6000 microscope (Leica (Wetzlar, Germany)).

### 4.6. Mitochondrial Morphology

Glass coverslips stained immunofluorescent for OXPHOS, cTnT and DAPI were imaged using a Leica Sp8 confocal microscope (Leica (Wetzlar, Germany)). Z stacks were obtained from three independent channels and imaging deconvolution was performed using Huygens Pro^®®^ (SVI (Hilversum, The Netherlands). Processed images were analyzed by Imares software^®®^ (Oxford instruments (Abingdon, UK). The cell surface was measured and 3D reconstruction was performed by surface area detailed level (0.2 µM) from blue (DAPI) and green channels (OXPHOS), respectively. The number of particles and total mitochondrial volume was quantified per cell.

### 4.7. Luciferase Viability Assay

Cell viability assays were performed using RealTime Glo MT Cell Viability Assay (Promega (Madison, WI, USA), #G9711), a non-lytic NanoLuc Luciferase reaction, according to the manufacturer’s instructions. Briefly, cardiomyocytes were seeded and cultured in sterile black-walled 96-well plates, 65,000 cells/well. 100 µL of RealTime-Glo reagent was added to the cells. After a 15 min incubation at 37 °C, the luminescence was recorded in a Synergy H1 microplate reader (Biotek (Winooski, VT, USA)) with an integration time of 1 s per well. For each condition the signal intensity was normalized to the total amount of protein per well, using DC protein assay kit (Biorad (Hercules, CA, USA), #500-0114).

### 4.8. Reactive Oxygen Species (ROS) Detection

ROS were detected with the cell-permeable, peroxide-sensitive fluorophore CellROX™ Orange reagent (Thermo Fisher Scientific (Waltham, MA, USA)) according to the manufacturer’s instructions. The dye is non-fluorescent while in a reduced state and exhibits bright orange fluorescence upon oxidation by ROS, with absorption/emission maxima of ~545/565 nm. In short, the hPSC-CMs were plated (50k cells/well) in Geltrex-coated blackwall 96-well plates. For measurement, the hPSC-CMs were incubated in a culture medium with 5 μM CellROX Orange reagent at 37 °C for 30 min, followed by washing twice with prewarmed PBS. End-point absorbance was then measured using a Synergy H1 microplate reader (BioTek (Winooski, VT, USA)). Measurements for CellROX Orange were normalized for protein content of each corresponding well. Protein concentration was measured with Detergent Compatible protein assay (Bio-rad (Hercules, CA, USA)) according to the manufacturer’s instructions. End-point absorbance (750 nm) was measured using the Synergy H1 microplate reader (BioTek (Winooski, VT, USA)).

### 4.9. Seahorse (Mitochondrial Respiration Assay)

The hPSC-CMs were seeded in 24-well Seahorse assay plates at a density of 100,000 cells/well. Mitochondrial function was determined by means of a Mito Stress test. Briefly, 1 h prior to the assay, the medium was replaced with an XF assay medium (102365-100, Agilent (Santa Clara, CA, USA)) supplemented with 10 mM glucose and 1 mM sodium pyruvate and the hPSC-CMs were incubated at 37 °C without CO_2_. After 3 baseline measurements, the ATP synthase inhibitor oligomycin A (1 μM; 75351, Sigma-Aldrich (St. Louis, MO, USA)) was injected, followed by subsequent injection of the uncoupler FCCP (0.5 μM; C2920, Sigma-Aldrich), and complex I and III inhibitors rotenone (1 μM; R8875, Sigma-Aldrich (St. Louis, MO, USA)) and antimycin A (1 μM; A8674, Sigma-Aldrich (St. Louis, MO, USA)), respectively. Cellular respiration was measured on a Seahorse XF24-3 Analyzer. Oxygen consumption rate (OCR) was normalized for total protein in each well. ATP synthase-linked (ATP-linked) respiration was calculated as the fraction of basal OCR minus the inhibited OCR after oligomycin A addition (OCR_basal_ − OCR_oligomycin A_; i.e., respiration dedicated to the production of ATP). Respiratory reserve was calculated as the capacity of cells to induce OCR beyond basal respiration (OCR_FCCP_ − OCR_basal_).

### 4.10. Mitochondrial Membrane Potential and Calcium Content

For mitochondrial potential measurements, hPSC-CMs plated in individual petridishes with glass bottoms (35 mm) were used 1 day after the medium change. Tetramethylrhodamine (TMRE; 1200 nM; T669, ThermoFisher (Waltham, MA, USA)) and Mitotracker Green (MTG; 100 nM; M7514; ThermoFisher (Waltham, MA, USA)) were added to the culture media and incubated for 30 min at 37 °C, 5% CO_2_. Hereafter, the hPSC-CMs were washed 3 times with CDM3. A time-lapse (200 pictures in 12 min) was made using with a Leica AF-6000 epifluorescence microscope (Leica (Wetzlar, Germany)). After 1 min, Rotenone (4 µM) and Antomycin-A (2 µM) were added to the CDM3 medium. One min before the end of the time-lapse, (20 µM) were added. Imaging analyses were performed using ImageJ.

For Mitochondrial calcium release, hPSC-CMs plated in individual Petri dishes with glass bottoms (35 mm) were used 1 day after the medium change. Fluo4-AM (5 µM; F14201; ThermoFisher (Waltham, MA, USA)) and Thapsigargine (10 µM; BML-PE180-001; Enzo life sciences (Bruxelles, Belgium) were added to the culture media and incubated for 230 min at 37 °C, 5% CO_2_. Hereafter, the hPSC-CMs were washed 3 times with Ca^2+^-free Krebs buffer. A time-lapse (200 pictures in 30 min) was made using with a Leica AF-6000 microscope. After 2 min, FCCP (20 µM) suspended in Krebs buffer was added. Imaging analyses were performed using ImageJ.

### 4.11. Statistical Analyses

Experimental groups consisted of at least 3 biological replicates and technical duplicates were used, unless stated differently in the manuscript. Data shown is representative for at least 3 independent experiments and is expressed as means ± standard error of the mean (SEM). The differences between the two groups were assessed by Student’s *t*-test using GraphPad Prism 7.02. A value of *p* < 0.05 was considered statistically significant.

## 5. Conclusions

We recognized DIO2 as a member of the cardiac fetal gene program in HF. DIO2 is responsible for tissue-specific transformation of inactive (T_4_) into active thyroid hormone (T_3_); a key driver of cardiac hypertrophy. We are the first to show in human stem cell derived cardiomyocytes that impairment of DIO2 expression severely affects several facets of mitochondrial dynamics. These results suggest a key position of human DIO2 and thyroid hormone homeostasis as a key regulator of mitochondrial function and bioenergetics in human cardiomyocytes and the development of heart failure.

## Data Availability

The data that support the findings of this study are available from the corresponding author upon reasonable request. RNA-seq data that support the findings in this study are available in the ArrayExpress database under accession number E-MTAB-5449.

## Supplementary Materials

The following are available online at https://www.mdpi.com/article/10.3390/ijms222111906/s1, Figure S1: Protein-protein interaction network visualization, Figure S2: Dio2 expression as result of cardiac disease in silico, in vivo and in vitro, Figure S3: Western blot results and analysis for TOM20 protein levels. Table S1: Overview of antibodies used for western blot and immunofluorescence, Table S2: Overview of primer sequences used for RT-qPCR, Table S3: Differentially expressed genes (DEG) in Ischemia/Reperfusion-induced heart failure, Table S4: DEGs inversely regulated with HF compared to a development stage, Supplementary Methods.

## Abbreviations

DIO2	Iodothyronine Deiodinase 2
UPRmt	Mitochondrial Unfolded Protein Response
LV	Left Ventricle
hPSC	human Pluripotent Stem Cells
hPSC-CMs	human Pluripotent Stem Cell-derived Cardiomyocytes
shDIO2	Short hairpin RNA-mediated specific silencing of DIO2
shSCR	Control short hairpin RNA-mediated silencing with a SCRambled sequence
HF	Heart Failure
ROS	Reactive Oxygen Species
FDR	False Discovery Rate
shRNA	shRNA: short hairpin RNA
IF	IF: Immunofluorescence
OCR	Oxygen Consumption Rate
DEG	Differentially Expressed Genes
GO-term	Gene Ontology-term
PPI	Protein-Protein Interaction
RT-qPCR	Real Time-quantitative Polymerase Chain Reaction
CAD	Coronary Artery Disease
GWA	Genome Wide Association
