# Supplementary material for: Selenoprotein DIO2 Is a Regulator of Mitochondrial Function, Morphology and UPRmt in Human Cardiomyocytes
Source: Int J Mol Sci. 2021 Nov 2;22(21):11906. doi: 10.3390/ijms222111906 (PMC8584701; doi:10.3390/ijms222111906)
Supplement: Supplementary file 1 [file ijms-22-11906-s001.zip › Supplemental figures_revised v3.pdf]

## SUPPLEMENTAL FIGURES:

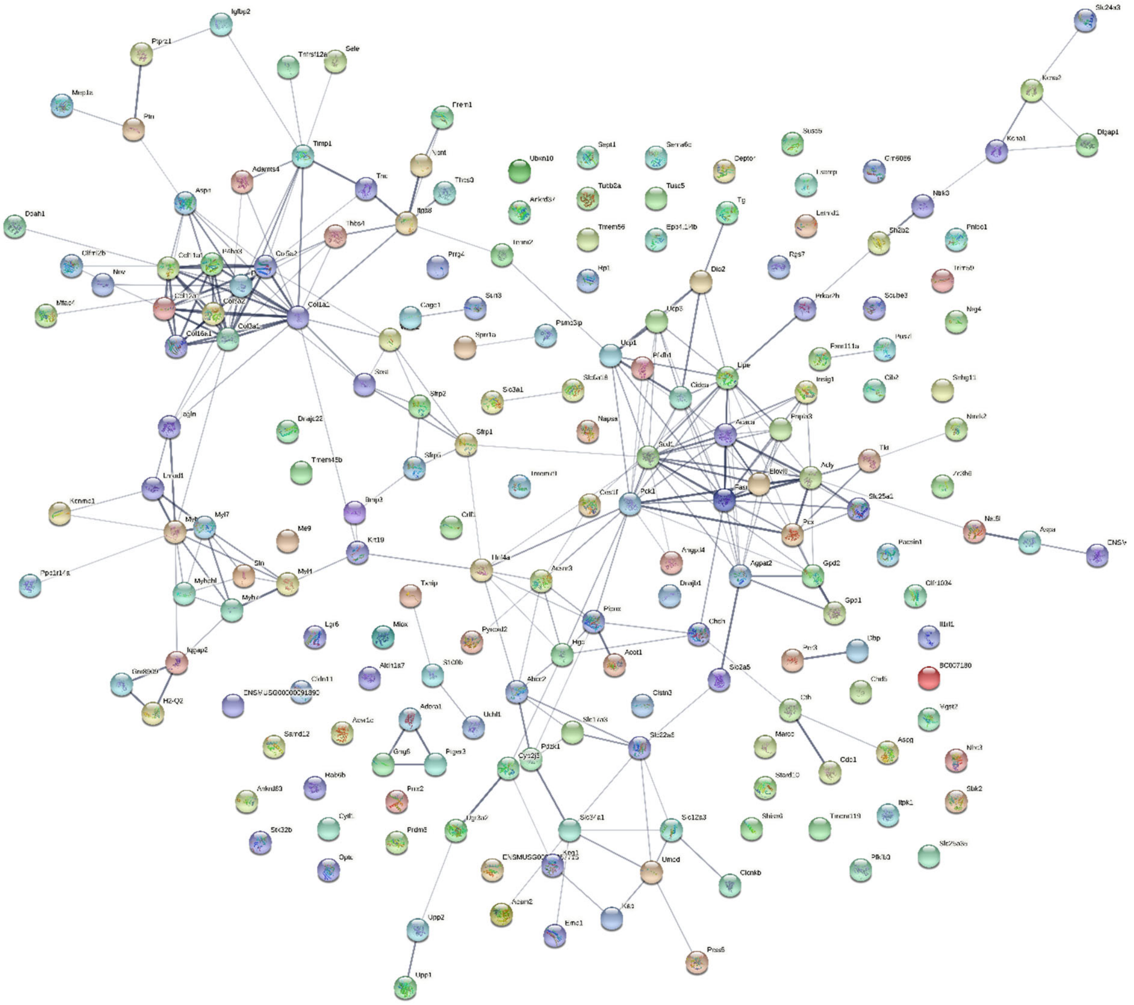

**Figure S1.** Visualization of the PPI-network (STRING-db). Protein-protein interactions (STRING-db) for de 242 differentially expressed genes (DEGs) contributing to the recapit-ulation of the fetal gene program.

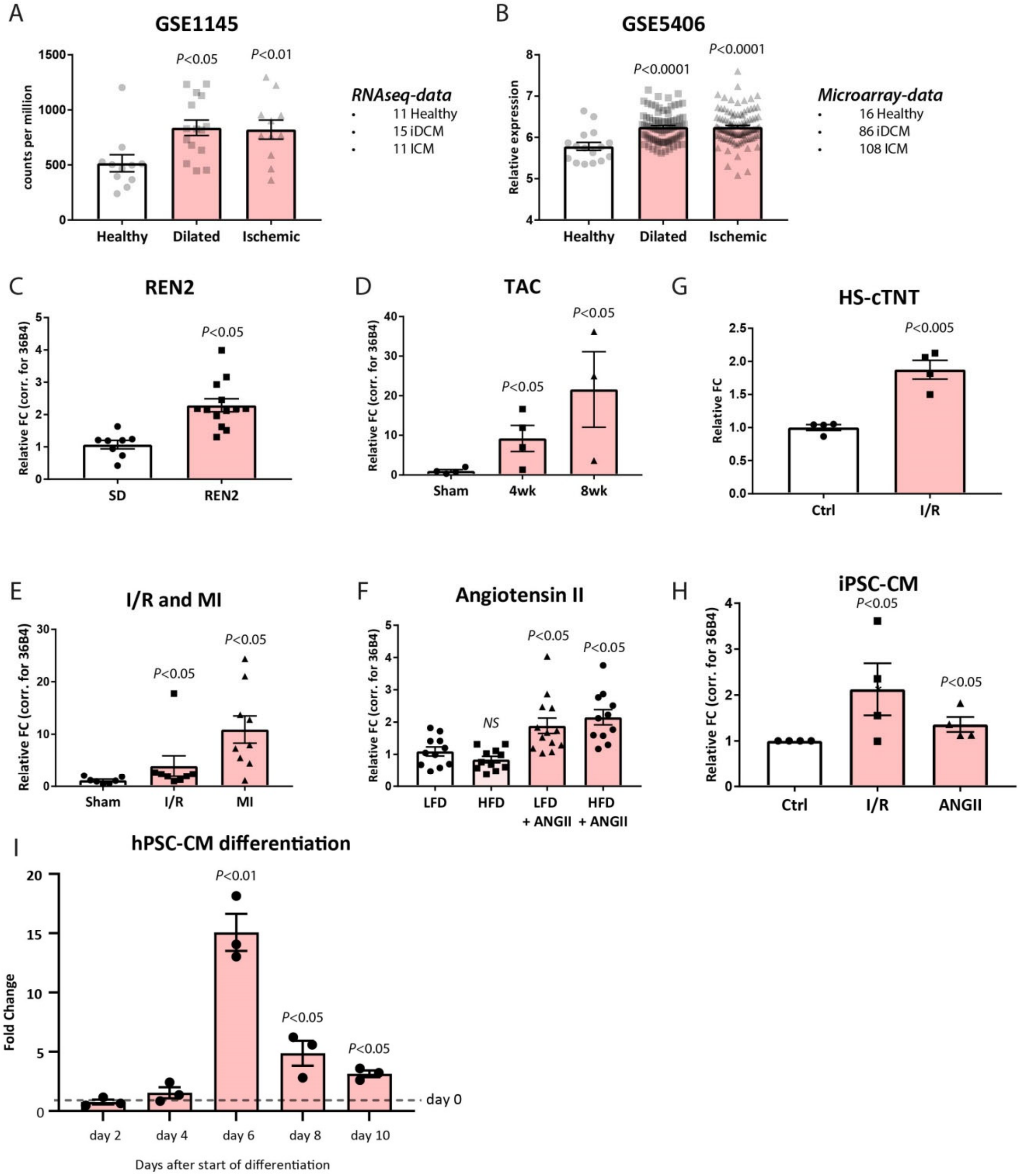


**Figure S2.** Dio2 expression as result of cardiac disease *in silico*, *in vivo* and *in vitro*. **(A)** *In silico* analysis of *Dio2* expression differences between 11 healthy, 15 idiopathic Dilated Cardiomyopathy (iDCM) and 11 Ischemic Cardiomyopathy (ICM) samples (GSE1145) and (B) 16 healthy, 86 iDCM and 108 ICM samples (GSE5406) showing significant induction of expression in iDCM and ICM samples. RT-qPCR analysis of Dio2 expression in several animal models **(C)** REN2 rats **(D)** TAC mice **(E)** Ischemia/Reperfusion (I/R) and Myocardial infarction (MI) injury and **(F)** Angiotension II infusion with Low Fat (LFD) or High Fat Diet (HFD). **(G)** High Sensitive cardiac Troponin T determination in culture medium of human pluripotent stem cell derived cardiomyocytes (hPSC-CM) that were exposed to subsequent 12 hour hypoxia/glucose depletion and 48 hours reoxygenation/glucose repletion (mimicking ischemia/reperfusion). **(H)** RT-qPCR analysis of Dio2 expression in hPSC-CM that were exposed to subsequent 12 hour hypoxia/glucose depletion and 48 hours reoxygenation/glucose repletion (mimicking ischemia/reperfusion (I/R)) and Angiotensin treatment (See supplemental methods). **(I)** RT-qPCR analysis of Dio2 expression in hPSC-CM during cardiomyocyte differentiation. mRNA expression on days 2-4-6-8 and 10 is relative to day 0 (start of differentiation, as described in the methods section).

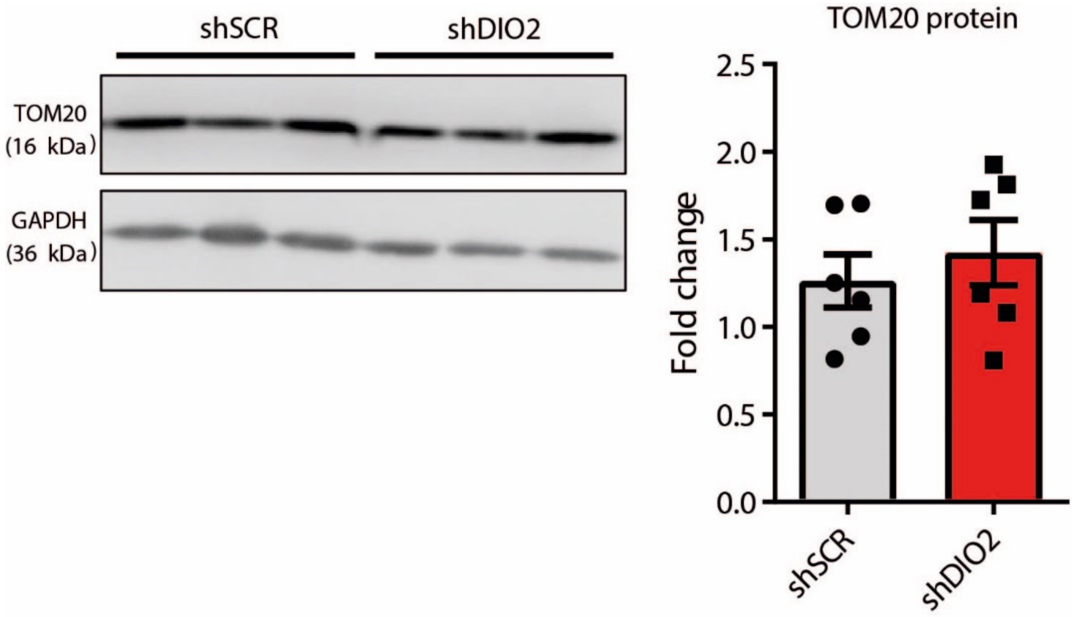


**Figure S3.** Western blot results and analysis for TOM20 protein levels (corrected for GAPDH)

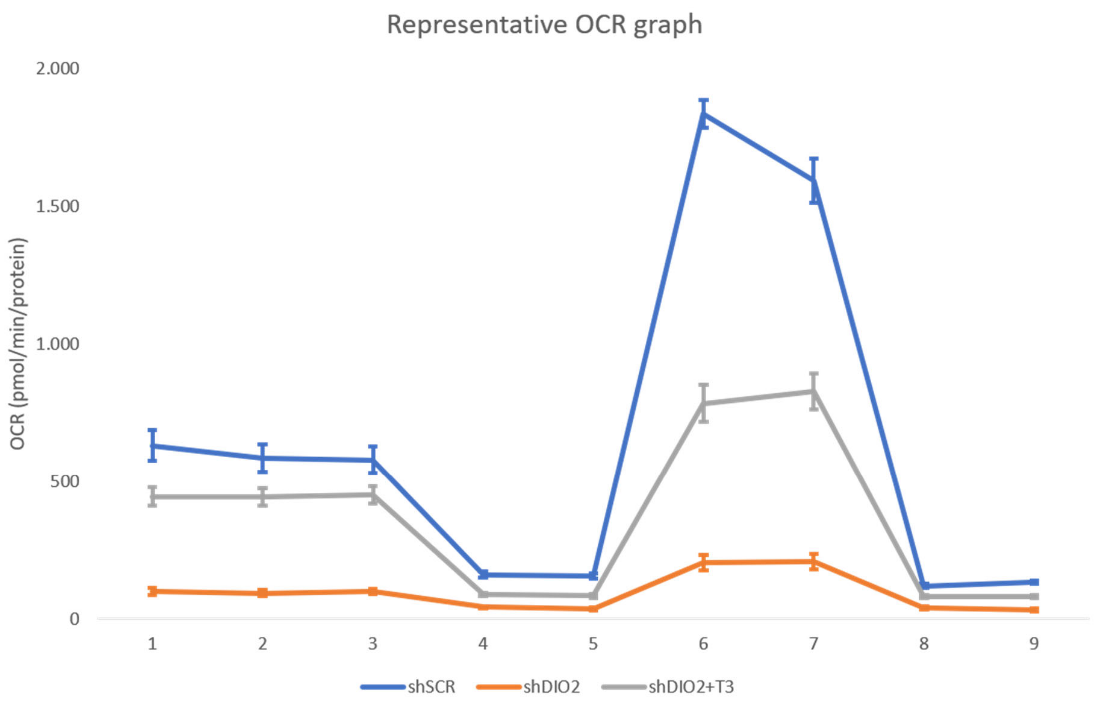


**Figure S4. Representative graph for mitochondrial respiration.** Oxygen Consumption rate (OCR) visualized for the conditions: shSCR (Blue line), shDIO2 (Orange line) and shDIO2 + T3 (Grey line). Values are plotted as mean±SE.
