# Supplementary material for: Selenoprotein DIO2 Is a Regulator of Mitochondrial Function, Morphology and UPRmt in Human Cardiomyocytes
Source: Int J Mol Sci. 2021 Nov 2;22(21):11906. doi: 10.3390/ijms222111906 (PMC8584701; doi:10.3390/ijms222111906)
Supplement: Supplementary file 1 [file ijms-22-11906-s001.zip › Supplemental methods_rebutal.pdf]

SUPPLEMENTAL MATERIALS AND METHODS:

Animal validation models

All animal experimental protocols were approved by the Animal Ethical Committee of the University of Groningen (permit numbers DEC6954A, DEC6827A, DEC6920A and IVD16487-03-01). All procedures were performed conform the existing guidelines for the care and use of laboratory animals. Male C57BL/6J mice (Envigo, the Netherlands) were housed on a 12/12 hours day/night cycle in a controlled environment and ad libitum access to water and chow. Mice were randomized based on bodyweight at an age of 8 weeks to create the experimental groups. Mice with abnormal bodyweight (>2 SD) or showing abnormal behavior were excluded. Prior to surgery, mice received a subcutaneous dose of carprofen (5.0mg/kg) to reduce post-operative wound pain.

REN2 (6954A)

Animal experiments were performed in 10-16 week-old male homozygous TGR (mREN2)27 rats (n=13). Age- and gender-matched Sprague Dawley (SD) rats (n=8) served as controls. REN2 rats overexpressed the murine Ren-2d gene that causes hypertension and progressive HF [Vernerova Z 2009, de Boer RA 2004, Groban L 2008]. The Ren2 and Sprague- Dawley rats were housed on a 12/12 hours day/night cycle in a controlled environment and ad libitum access to water and chow.

Myocardial Infarction (6827A)

Myocardial infarctions (MI) were induced as previously described.[24] In brief, mice were anesthetized, intubated and placed supine on a heated pad for mechanical ventilation. To create large MIs, the left anterior descending coronary artery (LAD) was permanently ligated using a suture. To create small MIs, a temporal ligation was performed. Hereby, a suture was tied onto a polyethylene tube placed on the LAD coronary artery and was removed again after 60 minutes. Control mice were sham operated.

TAC; cardiac pressure overload mouse model (6920A)

Mice underwent either a transverse aortic constriction (TAC) to generate cardiac pressure overload or were sham operated as a control, as described before [29]. In summary, mice were anesthetized with 2% isoflurane/oxygen, intubated and mechanically ventilated (Minivent, type 845, Harvard apparatus, USA) and placed on a heated pad to maintain adequate body temperature. After skin disinfection, a 0.5-1.0 cm incision was made to the chest. The thoracic cavity was opened between the second and third rib. Thereafter, a reproducible stenosis was created by tightening a 7-0 silk suture around the aortic arch between the brachiocephalic and left carotid arteries and a blunt 27G needle. After ligation, this needle was removed and the thoracic cavity and skin were closed using sutures. Sham procedures were identical except for the aortic arch ligation. After surgery, mice were placed in clean cages and chow (R70, Lantmännen, Sweden) with or without AZM198 was provided. To exclude differences in food intake between the two food types, food intake measurements were performed for a selection of animals for a period of 12 consecutive days. Mice were sacrificed at 4 or 8 weeks.

Obesity/hypertension mouse model (IVD16487-03-01)

Mice received a high fat diet (HFD) (60 kcal% fat, D12492, Research diets, USA), or a control low fat diet (LFD) (10 kcal% fat, D12450J, Research Diets, USA). Diets were substituted with 0.11% AZM198 in LFD or 0.16% AZM198 in HFD to obtain the same AZM198 target dose in both groups. As a control, no AZM198 was added. To exclude differences in food intake between experimental groups, food intake was measured for a selection of animals of each group for a period of 12 consecutive days.

After 12 weeks of diet intervention, mice were randomized to receive 4 weeks of either human angiotensin II (AngII) (1mg/kg/day dissolved in saline, Bachem, Switzerland) or saline infusion using osmotic minipumps (Alzet 1004, Durect corporation, USA). Pumps were combined with polyetheretherketone tubing (Alzet PEEK tubing 0002612, Durect corporation, USA) to allow MRI measurements. Pump placement was performed as described before [30]. In brief, mice were anesthetized with 2% isoflurane/oxygen via inhalation and were placed in the prone position on a heating pad to maintain body temperature. A subcutaneous pocket was created in the right flank for pump insertion. After placing the pump, the skin was closed using a 5-0 silk suture. Mice were sacrificed at 16 weeks.

Human validation dataset

Normalized expression data for DIO2 was downloaded from GEO (GSE5406(1); 16 non-failing (NF); 83 idiopathic dilated cardiomyopathy (iDCM); 101 ischemic cardiomyopathy (ICM) , and GSE1145; 11 non-failing; 15 iDCM; 11 ICM). Analyses on the normalized data were performed using GraphPad Prism 7.02. Differences between two groups were assessed by Student’s t-test. A value of $P<0.05$ was considered statistically significant.

Cell culture and cardiac stress models

In vitro Ischemia-reperfusion was induced by changing medium to non-glucose containing basal medium and transferring the cells to a hypoxic environment utilizing the BD GasPak EZ Anaerobe Pouch system. After 24 hours the cells are reintroduced to normoxic conditions and medium was changed back to CDM3 for 48 hours after which RNA and protein was isolated and media sampled. Troponin T levels were analysed using an electrochemiluminescence immunoassay kit (0509277, Roche Diagnostics). To induce hypertension-like stress in hPSC-CMs, 1 uM human angiotensin II (AngII) was added to the culture medium 48 hrs prior to isolation of RNA.

1. Hannenhalli S., Putt ME., Gilmore JM., et al. Transcriptional genomics associates FOX transcription factors with human heart failure. Circulation **2006**, 114(12):1269–76. Doi: 10.1161/CIRCULATIONAHA.106.632430.
