# Supplementary material for: Selenoprotein DIO2 Is a Regulator of Mitochondrial Function, Morphology and UPRmt in Human Cardiomyocytes
Source: Int J Mol Sci. 2021 Nov 2;22(21):11906. doi: 10.3390/ijms222111906 (PMC8584701; doi:10.3390/ijms222111906)
Supplement: Supplementary file 1 [file ijms-22-11906-s001.zip › Supplemental tables_rebutal.pdf]

Supplemental table S1:

Protein	Company	Cat. number
**DIO2**	Abcam	ab77779
**AKT**	Cell Signaling	4691
**pAKT**	Cell Signaling	4060
**AMPK**	Cell Signaling	2532
**pAMPK**	Cell Signaling	2535
**DNM1L**	Cell Signalling	5391
**pDNM1L**	Cell Signalling	4494
**MFN2**	Cell Signalling	11925
**OXPHOS**	Abcam	ab110413
**mtHSP70**	Thermo Fisher	MA3-028
**TOM20**	Cell Signaling	42406
**FoxO1**	Cell Signaling	2880
**pFoxO1**	Cell Signaling	9461
**p38-MAPK**	Cell Signaling	9212
**p-p38-MAPK**	Cell Signaling	9211S
**cleaved-CASP3**	Cell Signaling	9664
**CASP3**	Cell Signaling	9665
**Tubulin**	Sigma	t5168
**GAPDH**	Fitzgerald	10r-g109a

Supplemental table S2:

Primer	Forward primer 5' to 3'	Reverse primer 5' to 3'
**36B4**	AACGGGTACAAACGAGTC	AGATGGATCAGCCAAGAAG
**DIO2**	TTCCAGTGTGGTGCATGTCTC	AGTCAAGAAGGTGGCATGTGG
**DIO3**	GCACTTGGTTGGAACGCTATG	CAAGCGTGACTTGGTTTGAGG
**COL1A1**	GCCTCAAGGTATTGCTGGAC	ACCTTGTTTGCCAGGTTCAC
**PLN**	ACAGCTGCCAAGGCTACCTA	TCCATGATACCAGCAGGACA
**SERCA2a**	CGAACCCTTGCCACTCATCT	CCAGTATTGCAGGTTCCAGGT
**MYH6**	GATAGAGAGACTCCTGCGGC	TCGGTCATCTTGGTGCTTCC
**MYH7**	CGAAGGGCTTGAATGAGGAGT	TCCTCCCAAGGAGCTGTTAC
**MMP2**	CCCCAAAACGGACAAAGAG	CACGAGCAAAGGCATCATCC
**PGC-1α**	GCTGACAGATGGAGACGTGA	TAGCTGAGTGTTGGCTGGTG
**PGC-1β**	CGCTTTGAAGTGTTTGGTGAGATTG	GCTGGAAGGAGGGCTCGTTG
**PPARα**	CCAGTGGAGCATTGAACATC	TCGCACTTGTCATACACCAG
**ERRα**	CTCAGCTCCCTGCCCAAGCGC	CCGCTTGGTGATCTCACACTC
**ACACA**	AGAGGGAACATCCCTACGCT	CGAAAAGAGACCATTCCGCC
**ACACB**	TGAGTGGCTTTTGTCTGCCA	GCGGACAGACTTCTCCACAG
**ACLY**	GATTTTGCGGGGTTCGTCG	TTGCCCGTCTGCTCTGAAAT
**LDHA**	CAGCATAGCTGTTCCACTTAAGGC	GTTGCCATATTGGACTTGGAACC
**PKM**	GTGGGGCCATAATCGTCCTC	GACGAGCTGTCTGGGGATTC
**GLUT4**	TAGGCTCCGAAGATGGGGAA	CCCAGCCACGTCTCATTGTA
**FASN**	GACACAGTCACCATCTCGGG	GCGATGGCCTCCATGAAGTA
**MFN2**	ATGCATCCCCACTTAAGCAC	CCAGAGGGCAGAACTTTGTC
**FIS1**	TACGTCCGCGGGTTGCT	CCAGTTCCTTGGCCTGGTT
**OPA1**	TCAAGAAAAACTTGATGCTTTCA	GCAGAGCTGATTATGAGTACGATT
**DNM1L**	CCAAGGTGCCTGTAGGTGAT	CAGCAGTGACAGCGAGGATA
**GDF15**	GTGTTGCTGGTGCTCTCGTG	CGGTGTTCGAATCTTCCCAG
**ATF4**	CTATACCCAACAGGGCATCC	GTCCCTCCAACAACAGCAAG
**CHOP**	GACCTGCAAGAGGTCCTGTC	CAGTCAGCCAAGCCAGAGAA
**XBP1s**	CCAAGCGCTGTCTTAACTCC	GTGAGCTGGAACAGCAAGTG
**BiP**	TGATTGTCTTTTGTCAGGGGT	CACAGTGGTGCCTACCAAGA
**PDIA5**	TGCAGAGGACAGCCATGA	AGTGGAGAAAGGAGCCAGC
**GADD34**	GACTGCAAAGGCGGCTCAAG	TGCCCAGACAGCCAGGAAATG
**PDK4**	GTACAGTTGACCCAGTCACCA	TGGACCACTGCTACCACATC

Supplemental table S3:

EnsemblID	GeneName	log2 CPM PN20	log2 CPM HF20	log2FC HF20/PN20	FDR HF20/PN20
ENSMUSG00000091705	H2-Q2	-1,507	2,078	7,609	0,000
ENSMUSG00000067149	Jchain	-0,041	4,767	5,500	0,000
ENSMUSG00000076609	Igkc	2,161	7,304	5,263	0,000
ENSMUSG00000091345	Col6a5	0,810	4,673	4,240	0,008
ENSMUSG00000026390	Marco	-1,085	1,579	4,226	0,008
ENSMUSG00000073402	Gm8909	-0,919	1,782	3,964	0,049
ENSMUSG00000037625	Cldn11	-1,284	0,379	3,689	0,016
ENSMUSG00000030703	Gdpd3	1,502	4,940	3,597	0,000
ENSMUSG00000074280	Gm6166	-0,765	1,693	3,531	0,000
ENSMUSG00000027966	Col11a1	-0,405	2,348	3,508	0,002
ENSMUSG00000042254	Cilp	4,179	7,685	3,507	0,001
ENSMUSG00000076617	Ighm	4,208	7,504	3,318	0,001
ENSMUSG00000044042	Fmn1	2,554	5,808	3,313	0,000
ENSMUSG00000021702	Thbs4	3,135	6,377	3,274	0,000
ENSMUSG00000053093	Myh7	8,088	11,245	3,158	0,000
ENSMUSG00000038508	Gdf15	0,548	3,278	3,059	0,000
ENSMUSG00000097467	Gm26737	-1,095	0,456	3,001	0,010
ENSMUSG00000040985	Sun3	-0,173	2,215	2,982	0,000
ENSMUSG00000095079	Igha	1,432	4,229	2,957	0,000
ENSMUSG00000050359	Sprr1a	-0,771	1,165	2,954	0,000
ENSMUSG00000102189	Gm37194	0,776	3,441	2,931	0,000
ENSMUSG00000051048	P4ha3	-0,771	0,870	2,616	0,009
ENSMUSG00000056174	Col8a2	1,308	3,767	2,584	0,004
ENSMUSG00000007888	Crlf1	0,386	2,538	2,466	0,008
ENSMUSG00000004939	Nmrk2	1,676	3,881	2,329	0,000
ENSMUSG00000030329	Pianp	1,185	3,285	2,285	0,000
ENSMUSG00000027996	Sfrp2	3,092	5,256	2,223	0,009
ENSMUSG00000045967	Gpr158	0,051	1,851	2,219	0,000
ENSMUSG00000038274	Fau	-0,390	1,161	2,190	0,008
ENSMUSG00000028989	Angptl7	2,129	4,209	2,176	0,018
ENSMUSG00000028364	Tnc	1,681	3,658	2,097	0,001
ENSMUSG00000043999	Gpr75	0,077	1,749	2,094	0,001
ENSMUSG00000039476	Prrx2	-0,648	0,631	2,043	0,046
ENSMUSG00000001131	Timp1	1,485	3,382	2,035	0,008
ENSMUSG00000042436	Mfap4	4,678	6,678	2,018	0,006
ENSMUSG00000030862	Cpxm2	4,835	6,796	1,975	0,000
ENSMUSG00000029838	Ptn	2,317	4,204	1,938	0,007
ENSMUSG00000096403	Gm9825	-0,294	1,088	1,935	0,010
ENSMUSG00000032332	Col12a1	2,807	4,669	1,919	0,043
ENSMUSG00000036040	Adamtsl2	3,810	5,647	1,861	0,000
ENSMUSG00000054675	Tmem119	2,224	3,983	1,858	0,007
ENSMUSG00000097993	Ptprv	0,295	1,804	1,840	0,021

ENSMUSG00000031972	Acta1	8,027	9,861	1,835	0,000
ENSMUSG00000030762	Aqp8	1,705	3,432	1,822	0,012
ENSMUSG00000020911	Krt19	1,338	2,969	1,820	0,049
ENSMUSG00000047420	Fam180a	0,076	1,499	1,815	0,031
ENSMUSG00000029019	Nppb	6,741	8,537	1,800	0,000
ENSMUSG00000007682	Dio2	2,491	4,184	1,758	0,022
ENSMUSG00000063594	Gng8	0,369	1,798	1,734	0,020
ENSMUSG00000030116	Mfap5	4,437	6,150	1,726	0,005
ENSMUSG00000027656	Wisp2	3,674	5,356	1,710	0,008
ENSMUSG00000027750	Postn	6,818	8,519	1,703	0,016
ENSMUSG00000059049	Frem1	0,493	1,912	1,690	0,033
ENSMUSG00000105096	Gbp10	0,430	1,786	1,647	0,030
ENSMUSG00000032925	Itgbl1	4,549	6,110	1,572	0,015
ENSMUSG00000006403	Adamts4	1,563	2,972	1,520	0,000
ENSMUSG00000040276	Pacsin1	0,061	1,200	1,492	0,048
ENSMUSG00000081824	BC002163	1,498	2,866	1,485	0,000
ENSMUSG00000073437	D330041H03Rik	1,733	3,044	1,424	0,000
ENSMUSG00000091971	Hspa1a	0,802	2,022	1,423	0,007
ENSMUSG00000068196	Col8a1	5,742	7,141	1,404	0,008
ENSMUSG00000000486	Sept1	1,275	2,529	1,398	0,001
ENSMUSG00000044566	Cage1	0,692	1,866	1,390	0,031
ENSMUSG00000024011	Pi16	6,111	7,494	1,388	0,003
ENSMUSG00000001493	Meox1	4,339	5,704	1,381	0,001
ENSMUSG00000021388	Aspn	5,472	6,833	1,368	0,001
ENSMUSG00000060224	Pyroxd2	2,273	3,558	1,355	0,006
ENSMUSG00000019303	Psmc3ip	1,434	2,655	1,345	0,020
ENSMUSG00000000693	Loxl3	3,424	4,732	1,338	0,023
ENSMUSG00000031595	Pdgfrl	2,498	3,762	1,320	0,049
ENSMUSG00000020205	Phlda1	4,598	5,876	1,294	0,003
ENSMUSG00000021676	Iqgap2	3,390	4,627	1,264	0,035
ENSMUSG00000022951	Rcan1	6,648	7,906	1,262	0,000
ENSMUSG00000032322	Pstpip1	1,584	2,722	1,254	0,001
ENSMUSG00000023439	Gnb3	3,251	4,435	1,218	0,000
ENSMUSG00000023905	Tnfrsf12a	5,176	6,383	1,215	0,000
ENSMUSG00000020407	Upp1	1,919	3,066	1,210	0,000
ENSMUSG00000030208	Emp1	6,405	7,605	1,204	0,000
ENSMUSG00000042379	Esm1	1,260	2,328	1,201	0,009
ENSMUSG00000026043	Col3a1	9,019	10,216	1,197	0,003
ENSMUSG00000038463	Olfml2b	4,138	5,313	1,193	0,002
ENSMUSG00000005124	Wisp1	1,681	2,725	1,145	0,031
ENSMUSG00000033182	Kbtbd12	4,833	5,966	1,143	0,006
ENSMUSG00000034317	Trim59	1,076	2,045	1,120	0,050
ENSMUSG00000028194	Ddah1	3,522	4,600	1,113	0,001
ENSMUSG00000049871	Nlrc3	2,329	3,369	1,107	0,003
ENSMUSG00000103476	Gm34302	2,010	3,053	1,101	0,002
ENSMUSG00000040690	Col16a1	4,312	5,398	1,100	0,013

ENSMUSG00000026042	Col5a2	6,221	7,309	1,092	0,008
ENSMUSG00000058672	Tubb2a	3,714	4,780	1,082	0,000
ENSMUSG00000031548	Sfrp1	4,978	6,031	1,063	0,040
ENSMUSG00000024803	Ankrd1	10,091	11,153	1,062	0,018
ENSMUSG00000053469	Tg	1,745	2,696	1,046	0,030
ENSMUSG00000024691	Fam111a	3,690	4,703	1,044	0,000
ENSMUSG00000001506	Col1a1	7,886	8,928	1,043	0,050
ENSMUSG00000029223	Uchl1	3,005	4,007	1,039	0,043
ENSMUSG00000033356	Pus7l	1,510	2,430	1,039	0,026
ENSMUSG00000031740	Mmp2	6,628	7,651	1,026	0,026
ENSMUSG00000028047	Thbs3	3,496	4,496	1,025	0,022
ENSMUSG00000005483	Dnajb1	4,279	5,285	1,019	0,000
ENSMUSG00000036446	Lum	6,645	7,654	1,010	0,030
ENSMUSG00000050914	Ankrd37	1,603	2,498	1,010	0,022
ENSMUSG00000078190	Dnm3os	2,651	3,612	1,007	0,050
ENSMUSG00000028957	Per3	6,084	5,085	-1,003	0,027
ENSMUSG00000038777	Sema6c	3,922	2,942	-1,005	0,002
ENSMUSG00000028434	Epb41l4b	2,833	1,880	-1,016	0,011
ENSMUSG00000067818	Myl9	6,225	5,210	-1,019	0,029
ENSMUSG00000026582	Sele	2,160	1,199	-1,019	0,042
ENSMUSG00000103593	Gm37352	2,895	1,923	-1,020	0,005
ENSMUSG00000057963	Itpk1	4,686	3,675	-1,021	0,042
ENSMUSG00000030688	Stard10	5,165	4,142	-1,030	0,035
ENSMUSG00000045294	Insig1	5,344	4,321	-1,031	0,010
ENSMUSG00000032064	Dixdc1	4,706	3,685	-1,033	0,013
ENSMUSG00000043993	2900052L18Rik	2,133	1,173	-1,037	0,040
ENSMUSG00000018740	Slc25a35	4,544	3,525	-1,038	0,005
ENSMUSG00000042793	Lgr6	7,137	6,099	-1,041	0,000
ENSMUSG00000034926	Dhcr24	3,386	2,381	-1,045	0,009
ENSMUSG00000042851	Zc3h6	4,337	3,306	-1,047	0,000
ENSMUSG00000042429	Adora1	5,090	4,050	-1,049	0,014
ENSMUSG00000038393	Txnip	10,392	9,339	-1,053	0,000
ENSMUSG00000021379	Id4	1,961	0,993	-1,053	0,041
ENSMUSG00000046618	Olfml2a	4,610	3,551	-1,070	0,001
ENSMUSG00000051224	Tceanc	2,437	1,428	-1,079	0,012
ENSMUSG00000035783	Acta2	8,270	7,189	-1,082	0,001
ENSMUSG00000024892	Pcx	6,925	5,842	-1,085	0,015
ENSMUSG00000104346	Pcdhga3	2,439	1,410	-1,103	0,014
ENSMUSG00000105597	4633401B06Rik	2,653	1,606	-1,105	0,011
ENSMUSG00000073608	Gm6086	4,029	2,951	-1,106	0,000
ENSMUSG00000025453	Nnt	2,005	0,972	-1,114	0,038
ENSMUSG00000026922	Agpat2	6,978	5,865	-1,115	0,029
ENSMUSG00000101609	Kcnq1ot1	4,800	3,701	-1,116	0,000
ENSMUSG00000097284	4930480K23Rik	2,986	1,922	-1,127	0,005
ENSMUSG00000092341	Malat1	12,588	11,450	-1,138	0,000
ENSMUSG00000026839	Upp2	1,798	0,767	-1,142	0,049

ENSMUSG00000031938	4931406C07Rik	5,448	4,310	-1,142	0,039
ENSMUSG00000102440	Pcdhga9	1,802	0,771	-1,143	0,048
ENSMUSG00000053414	Hunk	1,858	0,821	-1,148	0,033
ENSMUSG00000024298	Zfp871	5,385	4,235	-1,158	0,000
ENSMUSG00000074024	4632427E13Rik	2,867	1,774	-1,162	0,002
ENSMUSG00000094410	Gm38394	2,928	1,814	-1,166	0,002
ENSMUSG00000006216	Clcnkb	2,099	1,008	-1,169	0,021
ENSMUSG00000067199	Frat1	2,453	1,353	-1,181	0,008
ENSMUSG00000008307	1700109H08Rik	2,719	1,606	-1,183	0,001
ENSMUSG00000043621	Ubxn10	2,283	1,181	-1,185	0,022
ENSMUSG00000037166	Ppp1r14a	3,237	2,097	-1,185	0,003
ENSMUSG00000107624	RP23-170C11.3	2,556	1,435	-1,191	0,029
ENSMUSG00000091890	A830073O21Rik	1,873	0,790	-1,197	0,046
ENSMUSG00000097877	Gm26703	1,662	0,555	-1,233	0,049
ENSMUSG00000102555	6430511E19Rik	1,892	0,779	-1,242	0,021
ENSMUSG00000034731	Dgkh	3,253	2,067	-1,245	0,000
ENSMUSG00000017453	Pipox	2,035	0,865	-1,266	0,027
ENSMUSG00000022419	Deptor	6,496	5,226	-1,273	0,009
ENSMUSG00000102336	Gm37233	2,367	1,173	-1,286	0,004
ENSMUSG00000089901	Gm8113	1,497	0,355	-1,289	0,041
ENSMUSG00000032942	Ucp3	7,090	5,804	-1,290	0,020
ENSMUSG00000102091	Olfr1034	1,709	0,555	-1,290	0,038
ENSMUSG00000020774	Aspa	3,349	2,099	-1,295	0,017
ENSMUSG00000026069	Il1rl1	2,216	1,018	-1,296	0,016
ENSMUSG00000032085	Tagln	6,546	5,249	-1,303	0,004
ENSMUSG00000020155	Kcnmb1	2,712	1,481	-1,303	0,021
ENSMUSG00000097601	Gm26660	1,711	0,551	-1,305	0,022
ENSMUSG00000053930	Shisa6	2,611	1,389	-1,306	0,048
ENSMUSG00000102602	A930004J17Rik	3,377	2,111	-1,322	0,000
ENSMUSG00000010492	Uckl1os	3,059	1,781	-1,334	0,001
ENSMUSG00000020182	Ddc	2,422	1,151	-1,346	0,001
ENSMUSG00000026605	Cenpf	5,637	4,298	-1,349	0,000
ENSMUSG00000059824	Dbp	6,748	5,398	-1,355	0,003
ENSMUSG00000040724	Kcna2	3,896	2,571	-1,355	0,020
ENSMUSG00000028076	Cd1d1	3,137	1,787	-1,388	0,034
ENSMUSG00000072949	Acot1	3,875	2,505	-1,411	0,004
ENSMUSG00000028132	Tmem56	2,893	1,543	-1,426	0,000
ENSMUSG00000029123	Stk32b	1,814	0,549	-1,430	0,011
ENSMUSG00000097536	2610037D02Rik	2,174	0,854	-1,445	0,001
ENSMUSG00000061080	Lsamp	3,440	2,030	-1,466	0,000
ENSMUSG00000102516	Gm38340	2,433	1,070	-1,469	0,021
ENSMUSG00000024112	Cacna1h	5,035	3,580	-1,471	0,002
ENSMUSG00000107742	RP23-89F9.2	1,883	0,553	-1,479	0,005
ENSMUSG00000047976	Kcna1	2,397	1,028	-1,482	0,008
ENSMUSG00000025271	Pfkfb1	5,399	3,921	-1,496	0,000
ENSMUSG00000026773	Pfkfb3	5,787	4,286	-1,508	0,016

ENSMUSG00000085558	4930412C18Rik	2,050	0,697	-1,514	0,006
ENSMUSG00000005045	Chd5	1,536	0,233	-1,520	0,022
ENSMUSG00000027171	Prrg4	1,822	0,452	-1,530	0,004
ENSMUSG00000058656	Samd12	1,147	-0,118	-1,533	0,048
ENSMUSG00000093672	Gm20655	1,167	-0,101	-1,535	0,040
ENSMUSG00000037353	Letmd1	5,704	4,159	-1,549	0,027
ENSMUSG00000108365	RP24-174G2.2	1,436	0,108	-1,561	0,018
ENSMUSG00000062329	Cytl1	4,337	2,805	-1,566	0,000
ENSMUSG00000028179	Cth	2,258	0,827	-1,570	0,008
ENSMUSG00000105962	Gm42432	2,372	0,924	-1,577	0,001
ENSMUSG00000024526	Cidea	6,724	5,144	-1,582	0,010
ENSMUSG00000030433	Sbk2	3,351	1,835	-1,582	0,001
ENSMUSG00000022490	Ppp1r1a	2,426	0,968	-1,585	0,004
ENSMUSG00000018486	Wnt9b	1,542	0,178	-1,587	0,028
ENSMUSG00000018830	Myh11	8,072	6,486	-1,588	0,000
ENSMUSG00000096954	Gdap10	4,184	2,625	-1,596	0,000
ENSMUSG00000102964	9430034N14Rik	1,543	0,174	-1,601	0,014
ENSMUSG00000103772	Gm36933	1,453	0,096	-1,605	0,025
ENSMUSG00000105572	Gm43300	1,468	0,101	-1,614	0,019
ENSMUSG00000026527	Rgs7	3,017	1,458	-1,618	0,042
ENSMUSG00000078161	Erich3	1,408	0,038	-1,619	0,015
ENSMUSG00000022613	Miox	1,866	0,415	-1,628	0,003
ENSMUSG00000106706	C530043K16Rik	1,144	-0,187	-1,639	0,029
ENSMUSG00000024650	Slc22a6	1,618	0,180	-1,644	0,006
ENSMUSG00000045968	Teddm2	1,501	0,113	-1,646	0,011
ENSMUSG00000103133	Gm37303	1,657	0,228	-1,652	0,004
ENSMUSG00000103674	Gm37699	1,256	-0,103	-1,654	0,018
ENSMUSG00000003123	Lipe	7,612	5,956	-1,659	0,000
ENSMUSG00000099931	Gm29358	1,453	0,038	-1,680	0,011
ENSMUSG00000036083	Slc17a3	1,732	0,231	-1,693	0,005
ENSMUSG00000073485	H3f3aos	1,904	0,406	-1,702	0,001
ENSMUSG00000104271	Gm37891	1,639	0,173	-1,705	0,005
ENSMUSG00000097324	Mir143hg	2,951	1,349	-1,721	0,000
ENSMUSG00000025900	Rp1	1,809	0,303	-1,725	0,004
ENSMUSG00000026676	Ccdc3	3,745	2,061	-1,731	0,012
ENSMUSG00000105556	Gm43080	3,000	1,371	-1,738	0,000
ENSMUSG00000052520	Cyp2j5	2,817	1,212	-1,743	0,003
ENSMUSG00000064371	mt-Tt	2,278	0,695	-1,744	0,001
ENSMUSG00000031665	Sall1	1,951	0,394	-1,753	0,001
ENSMUSG00000103585	Pcdhgb4	2,922	1,287	-1,754	0,000
ENSMUSG00000073460	Pnldc1	1,691	0,166	-1,755	0,033
ENSMUSG00000103897	Pcdhga8	1,360	-0,108	-1,774	0,008
ENSMUSG00000105199	Gm43581	1,183	-0,266	-1,798	0,035
ENSMUSG00000030108	Slc6a13	1,066	-0,362	-1,798	0,028
ENSMUSG00000025255	Zfhx4	2,849	1,146	-1,822	0,003
ENSMUSG00000003279	Dlgap1	1,596	0,038	-1,833	0,035

ENSMUSG00000088689	Scarna17	1,121	-0,349	-1,852	0,022
ENSMUSG00000107756	RP24-359O2.7	1,139	-0,357	-1,863	0,008
ENSMUSG00000031766	Slc12a3	2,073	0,456	-1,869	0,026
ENSMUSG00000032549	Rab6b	5,608	3,748	-1,873	0,028
ENSMUSG00000086916	Gm15903	1,727	0,101	-1,886	0,001
ENSMUSG00000024008	Cpne5	1,464	-0,101	-1,889	0,006
ENSMUSG00000017950	Hnf4a	1,695	0,110	-1,893	0,020
ENSMUSG00000107171	Gm42572	0,888	-0,549	-1,901	0,037
ENSMUSG00000038298	Pdzk1	2,424	0,743	-1,911	0,022
ENSMUSG00000002289	Angptl4	5,745	3,809	-1,956	0,000
ENSMUSG00000030945	Acsm2	4,095	2,226	-1,958	0,017
ENSMUSG00000106490	Gm43283	0,957	-0,549	-1,966	0,021
ENSMUSG00000002204	Napsa	4,020	2,153	-1,968	0,009
ENSMUSG00000026163	Sphkap	1,551	-0,106	-1,969	0,017
ENSMUSG00000061601	Pclo	1,818	0,113	-1,981	0,020
ENSMUSG00000021490	Slc34a1	4,316	2,389	-1,988	0,000
ENSMUSG00000032758	Kap	6,165	4,197	-1,991	0,004
ENSMUSG00000044349	Snhg11	2,712	0,892	-1,994	0,019
ENSMUSG00000103151	Gm38292	0,842	-0,646	-2,001	0,040
ENSMUSG00000049152	Ugt3a2	2,783	0,868	-2,010	0,000
ENSMUSG00000102856	Gm37084	2,758	0,894	-2,022	0,000
ENSMUSG00000104030	5330406M23Rik	1,685	-0,034	-2,035	0,000
ENSMUSG00000038677	Scube3	2,875	0,977	-2,041	0,013
ENSMUSG00000103123	Gm37390	0,878	-0,651	-2,044	0,021
ENSMUSG00000102153	Gm37474	1,393	-0,268	-2,045	0,002
ENSMUSG00000104094	Gm37314	1,506	-0,177	-2,048	0,006
ENSMUSG00000024694	Keg1	2,670	0,819	-2,051	0,001
ENSMUSG00000026827	Gpd2	6,711	4,661	-2,056	0,001
ENSMUSG00000022821	Hgd	1,229	-0,443	-2,093	0,005
ENSMUSG00000025094	Slc18a2	1,220	-0,440	-2,101	0,006
ENSMUSG00000025194	Abcc2	2,209	0,405	-2,110	0,020
ENSMUSG00000105434	Gm43359	3,314	1,329	-2,114	0,000
ENSMUSG00000105655	Gm42659	1,667	-0,111	-2,116	0,000
ENSMUSG00000044471	Lincpint	1,212	-0,448	-2,119	0,005
ENSMUSG00000032311	Nrg4	3,397	1,384	-2,120	0,000
ENSMUSG00000034009	Rxfp1	1,927	0,036	-2,145	0,048
ENSMUSG00000103313	Gm38357	0,587	-0,889	-2,154	0,041
ENSMUSG00000104388	Gm37033	3,483	1,432	-2,164	0,000
ENSMUSG00000086596	Susd5	3,288	1,247	-2,165	0,004
ENSMUSG00000101257	2310015K22Rik	0,613	-0,889	-2,173	0,034
ENSMUSG00000102352	Gm38346	1,395	-0,349	-2,175	0,002
ENSMUSG00000103780	Gm37524	0,807	-0,768	-2,180	0,025
ENSMUSG00000063873	Slc24a3	4,467	2,317	-2,199	0,000
ENSMUSG00000048142	Nat8l	4,506	2,336	-2,207	0,001
ENSMUSG00000104164	Gm38248	1,169	-0,546	-2,233	0,005
ENSMUSG00000023019	Gpd1	8,964	6,721	-2,245	0,004

ENSMUSG00000102594	Gm38381	0,870	-0,770	-2,251	0,011
ENSMUSG00000106121	Gm42679	1,193	-0,543	-2,269	0,005
ENSMUSG00000001420	Tmem79	2,688	0,558	-2,279	0,024
ENSMUSG00000103976	Gm37677	2,627	0,549	-2,295	0,000
ENSMUSG00000020917	Acly	8,650	6,354	-2,298	0,006
ENSMUSG00000003528	Slc25a1	6,168	3,876	-2,307	0,012
ENSMUSG00000005057	Sh2b2	3,476	1,223	-2,342	0,034
ENSMUSG00000106826	Gm42583	1,299	-0,543	-2,366	0,003
ENSMUSG00000103697	Gm38020	1,287	-0,551	-2,368	0,001
ENSMUSG00000048096	Lmod1	4,620	2,294	-2,388	0,000
ENSMUSG00000068748	Ptprz1	2,897	0,687	-2,401	0,000
ENSMUSG00000104012	Gm37364	0,293	-1,183	-2,428	0,042
ENSMUSG00000021613	Hapln1	0,320	-1,183	-2,439	0,041
ENSMUSG00000087382	Ctcflos	2,727	0,449	-2,457	0,028
ENSMUSG00000037362	Nov	4,695	2,296	-2,464	0,000
ENSMUSG00000026768	Itga8	6,068	3,609	-2,485	0,000
ENSMUSG00000102151	Gm37472	2,195	0,045	-2,499	0,000
ENSMUSG00000024131	Slc3a1	2,566	0,403	-2,500	0,007
ENSMUSG00000048040	Arxes2	2,042	-0,118	-2,506	0,006
ENSMUSG00000041653	Pnpla3	5,123	2,639	-2,515	0,022
ENSMUSG00000021612	Slc6a18	2,606	0,403	-2,519	0,001
ENSMUSG00000107813	RP23-92M3.1	0,658	-1,030	-2,533	0,011
ENSMUSG00000054966	Lmntd1	2,916	0,568	-2,544	0,002
ENSMUSG00000031725	Ces1f	2,963	0,639	-2,554	0,034
ENSMUSG00000103507	Gm38375	1,128	-0,773	-2,557	0,001
ENSMUSG00000021957	Tkt	8,245	5,686	-2,564	0,002
ENSMUSG00000023914	Mep1a	3,803	1,385	-2,582	0,001
ENSMUSG00000105395	Gm42672	0,726	-1,038	-2,612	0,006
ENSMUSG00000015970	Chdh	3,455	0,956	-2,653	0,035
ENSMUSG00000049336	Tenm2	0,763	-1,027	-2,662	0,005
ENSMUSG00000018822	Sfrp5	2,528	0,180	-2,676	0,000
ENSMUSG00000104339	C130089K02Rik	1,012	-0,897	-2,689	0,001
ENSMUSG00000105804	Gm43654	0,532	-1,180	-2,711	0,026
ENSMUSG00000074604	Mgst2	0,520	-1,183	-2,745	0,015
ENSMUSG00000107706	RP23-62G15.1	1,291	-0,765	-2,757	0,000
ENSMUSG00000001349	Cnn1	4,272	1,605	-2,779	0,000
ENSMUSG00000037493	Cib2	3,087	0,497	-2,788	0,010
ENSMUSG00000046275	Tusc5	5,389	2,635	-2,795	0,001
ENSMUSG00000033208	S100b	2,707	0,171	-2,802	0,005
ENSMUSG00000028976	Slc2a5	2,003	-0,359	-2,827	0,001
ENSMUSG00000105733	Gm42973	0,894	-1,030	-2,828	0,002
ENSMUSG00000040998	Npnt	4,391	1,659	-2,839	0,000
ENSMUSG00000030963	Umod	2,598	0,113	-2,877	0,000
ENSMUSG00000105445	Gm42972	0,939	-1,027	-2,884	0,001
ENSMUSG00000039323	Igfbp2	0,652	-1,183	-2,901	0,004
ENSMUSG00000030935	Acsm3	3,801	1,045	-2,902	0,003

ENSMUSG00000078137	Ankrd63	2,224	-0,261	-2,957	0,000
ENSMUSG00000030607	Acan	0,709	-1,183	-2,966	0,004
ENSMUSG00000026347	Tmem163	1,585	-0,770	-3,027	0,004
ENSMUSG00000105950	Gm43679	0,376	-1,354	-3,028	0,029
ENSMUSG00000026834	Acvr1c	2,791	0,082	-3,048	0,039
ENSMUSG00000001494	Sost	3,661	0,785	-3,060	0,000
ENSMUSG00000061718	Ppp1r1b	1,807	-0,654	-3,123	0,000
ENSMUSG00000040016	Ptger3	3,721	0,747	-3,183	0,029
ENSMUSG00000057715	A830018L16Rik	0,497	-1,351	-3,203	0,029
ENSMUSG00000102573	Gm7265	1,465	-0,894	-3,207	0,000
ENSMUSG00000033022	Cdo1	5,931	2,744	-3,245	0,006
ENSMUSG00000010311	Optc	1,528	-0,897	-3,283	0,000
ENSMUSG00000038009	Dnajc22	0,576	-1,351	-3,317	0,010
ENSMUSG00000105119	Gm43765	0,623	-1,351	-3,346	0,004
ENSMUSG00000020532	Acaca	6,908	3,546	-3,389	0,001
ENSMUSG00000030111	A2m	1,080	-1,183	-3,393	0,002
ENSMUSG00000037686	Aspg	3,352	0,226	-3,408	0,026
ENSMUSG00000093465	Gm20682	0,173	-1,548	-3,514	0,034
ENSMUSG00000106749	Gm43281	0,870	-1,354	-3,661	0,000
ENSMUSG00000041737	Tmem45b	4,485	0,931	-3,774	0,050
ENSMUSG00000030800	Prss8	1,477	-1,180	-3,893	0,000
ENSMUSG00000029335	Bmp3	1,538	-1,183	-3,911	0,006
ENSMUSG00000061086	Myl4	6,654	2,739	-3,966	0,000
ENSMUSG00000029671	Wnt16	0,521	-1,545	-4,021	0,004
ENSMUSG00000025153	Fasn	11,047	6,826	-4,226	0,000
ENSMUSG00000020891	Alox8	0,684	-1,545	-4,231	0,001
ENSMUSG00000002997	Prkar2b	6,015	1,833	-4,291	0,000
ENSMUSG00000069378	Prdm6	0,861	-1,548	-4,436	0,000
ENSMUSG00000024747	Aldh1a7	3,082	-0,665	-4,478	0,014
ENSMUSG00000037071	Scd1	11,477	6,979	-4,502	0,002
ENSMUSG00000041220	Elovl6	6,507	1,887	-4,720	0,000
ENSMUSG00000068745	Mybphl	3,876	-0,349	-4,756	0,000
ENSMUSG00000027513	Pck1	7,266	2,572	-4,770	0,000
ENSMUSG00000086843	E030013I19Rik	2,669	-1,033	-4,803	0,000
ENSMUSG00000059146	Ntrk3	5,083	0,546	-4,810	0,000
ENSMUSG00000002341	Ncan	0,312	-1,768	-5,472	0,012
ENSMUSG00000048355	Arxes1	0,321	-1,768	-5,542	0,004
ENSMUSG00000020469	Myl7	6,007	0,652	-5,604	0,000
ENSMUSG00000008153	Clstn3	3,711	-1,030	-5,817	0,000
ENSMUSG00000042045	Sln	4,347	-1,027	-6,475	0,000
ENSMUSG00000030046	Bmp10	4,184	-1,545	-7,954	0,000
ENSMUSG00000031710	Ucp1	7,069	-0,540	-8,354	0,000

Supplemental table S4:

EnsemblID	GeneName	log2FC PN20/EB12	FDR PN20/EB12	log2FC PN20/EB18	FDR PN20/EB18	log2FC PN20/PND2	FDR PN20/PND2	log2FC HF20/PN20	FDR HF20/PN20	GeneAnnotation
ENSMUSG00000031710	Ucp1	11,561	0,000	9,749	0,000	11,069	0,000	**-8,354**	**0,000**	uncoupling protein 1 (mitochondrial, proton carrier) [Source:MGI Symbol;Acc:MGI:98894]
ENSMUSG00000053093	Myh7	-4,022	0,000	-5,573	0,000	-1,860	0,000	**3,158**	**0,000**	myosin, heavy polypeptide 7, cardiac muscle, beta [Source:MGI Symbol;Acc:MGI:2155600]
ENSMUSG00000086843	E030013I19Rik	2,817	0,000	3,907	0,000	4,200	0,000	**-4,803**	**0,000**	RIKEN cDNA E030013I19 gene [Source:MGI Symbol;Acc:MGI:2443735]
ENSMUSG00000092341	Malat1	1,924	0,000	1,132	0,000	0,604	0,000	**-1,138**	**0,000**	metastasis associated lung adenocarcinoma transcript 1 (non-coding RNA) [Source:MGI Symbol;Acc:MGI:1919539]
ENSMUSG00000105434	Gm43359	0,806	0,002	-0,111	0,678	-1,013	0,000	**-2,114**	**0,000**	predicted gene 43359 [Source:MGI Symbol;Acc:MGI:5663496]
ENSMUSG00000002289	Angptl4	2,348	0,000	2,998	0,000	-0,212	0,514	**-1,956**	**0,000**	angiopoietin-like 4 [Source:MGI Symbol;Acc:MGI:1888999]
ENSMUSG00000018822	Sfrp5	0,306	0,352	0,626	0,042	1,231	0,000	**-2,676**	**0,000**	secreted frizzled-related sequence protein 5 [Source:MGI Symbol;Acc:MGI:1860298]
ENSMUSG00000040998	Npnt	-1,826	0,000	1,007	0,013	2,198	0,000	**-2,839**	**0,000**	nephronectin [Source:MGI Symbol;Acc:MGI:2148811]
ENSMUSG00000104388	Gm37033	2,962	0,000	1,798	0,000	1,539	0,000	**-2,164**	**0,000**	predicted gene, 37033 [Source:MGI Symbol;Acc:MGI:5610261]
ENSMUSG00000020469	Myl7	-2,738	0,000	-0,619	0,429	1,912	0,012	**-5,604**	**0,000**	myosin, light polypeptide 7, regulatory [Source:MGI Symbol;Acc:MGI:107495]
ENSMUSG00000078137	Ankrd63	0,893	0,013	-3,263	0,000	-1,330	0,000	**-2,957**	**0,000**	ankyrin repeat domain 63 [Source:MGI Symbol;Acc:MGI:2686183]
ENSMUSG00000048096	Lmod1	2,234	0,000	3,245	0,000	4,197	0,000	**-2,388**	**0,000**	leiomodin 1 (smooth muscle) [Source:MGI Symbol;Acc:MGI:2135671]
ENSMUSG00000049152	Ugt3a2	8,711	0,000	6,915	0,000	8,366	0,000	**-2,010**	**0,000**	UDP glycosyltransferases 3 family, polypeptide A2 [Source:MGI Symbol;Acc:MGI:2145969]
ENSMUSG00000096954	Gdap10	1,722	0,000	1,695	0,000	1,642	0,000	**-1,596**	**0,000**	ganglioside-induced differentiation-associated-protein 10 [Source:MGI Symbol;Acc:MGI:1338008]
ENSMUSG00000025271	Pfkfb1	2,158	0,000	3,035	0,000	2,978	0,000	**-1,496**	**0,000**	6-phosphofructo-2-kinase/fructose-2,6-biphosphatase 1 [Source:MGI Symbol;Acc:MGI:107816]

ENSMUSG00000102151	Gm37472	1,977	0,000	1,520	0,000	0,921	0,009	**-2,499**	**0,000**	predicted gene, 37472 [Source:MGI Symbol;Acc:MGI:5610700]
ENSMUSG00000105556	Gm43080	1,069	0,000	0,058	0,911	0,244	0,436	**-1,738**	**0,000**	predicted gene 43080 [Source:MGI Symbol;Acc:MGI:5663217]
ENSMUSG00000026768	Itga8	0,799	0,054	0,820	0,058	1,126	0,010	**-2,485**	**0,000**	integrin alpha 8 [Source:MGI Symbol;Acc:MGI:109442]
ENSMUSG00000062329	Cytl1	4,272	0,000	1,659	0,000	2,406	0,000	**-1,566**	**0,000**	cytokine-like 1 [Source:MGI Symbol;Acc:MGI:2684993]
ENSMUSG00000042045	Sln	4,152	0,000	1,879	0,038	4,329	0,000	**-6,475**	**0,000**	sarcolipin [Source:MGI Symbol;Acc:MGI:1913652]
ENSMUSG00000010311	Optc	2,298	0,000	3,742	0,000	4,592	0,000	**-3,283**	**0,000**	opticin [Source:MGI Symbol;Acc:MGI:2151113]
ENSMUSG00000097324	Mir143hg	1,624	0,000	2,137	0,000	0,507	0,101	**-1,721**	**0,000**	Mir143 and Mir145 host gene (non-protein coding) [Source:MGI Symbol;Acc:MGI:4439832]
ENSMUSG00000102856	Gm37084	0,790	0,011	0,286	0,404	0,225	0,557	**-2,022**	**0,000**	predicted gene, 37084 [Source:MGI Symbol;Acc:MGI:5610312]
ENSMUSG00000040985	Sun3	0,446	0,609	-1,373	0,048	-2,429	0,000	**2,982**	**0,000**	Sad1 and UNC84 domain containing 3 [Source:MGI Symbol;Acc:MGI:3041199]
ENSMUSG00000037362	Nov	3,463	0,000	0,955	0,032	0,086	0,894	**-2,464**	**0,000**	nephroblastoma overexpressed gene [Source:MGI Symbol;Acc:MGI:109185]
ENSMUSG00000042793	Lgr6	4,715	0,000	2,779	0,000	1,729	0,000	**-1,041**	**0,000**	leucine-rich repeat-containing G protein-coupled receptor 6 [Source:MGI Symbol;Acc:MGI:2441805]
ENSMUSG00000004939	Nmrk2	3,333	0,000	-1,548	0,000	-2,679	0,000	**2,329**	**0,000**	nicotinamide riboside kinase 2 [Source:MGI Symbol;Acc:MGI:1916814]
ENSMUSG00000032311	Nrg4	3,980	0,000	3,689	0,000	4,770	0,000	**-2,120**	**0,000**	neuregulin 4 [Source:MGI Symbol;Acc:MGI:1933833]
ENSMUSG00000030800	Prss8	-0,337	0,574	2,641	0,000	4,048	0,000	**-3,893**	**0,000**	protease, serine 8 (prostasin) [Source:MGI Symbol;Acc:MGI:1923810]
ENSMUSG00000021490	Slc34a1	10,377	0,000	10,227	0,000	10,032	0,000	**-1,988**	**0,000**	solute carrier family 34 (sodium phosphate), member 1 [Source:MGI Symbol;Acc:MGI:1345284]
ENSMUSG00000074280	Gm6166	-0,461	0,835	-2,177	0,007	-1,496	0,143	**3,531**	**0,000**	predicted gene 6166 [Source:MGI Symbol;Acc:MGI:3645893]
ENSMUSG00000059146	Ntrk3	0,822	0,318	2,542	0,003	2,890	0,001	**-4,810**	**0,000**	neurotrophic tyrosine kinase, receptor, type 3 [Source:MGI Symbol;Acc:MGI:97385]
ENSMUSG00000063873	Slc24a3	0,947	0,020	1,803	0,000	2,849	0,000	**-2,199**	**0,000**	solute carrier family 24 (sodium/potassium/calcium exchanger), member 3 [Source:MGI Symbol;Acc:MGI:2137513]

ENSMUSG00000061086	Myl4	-3,107	0,000	-1,861	0,013	1,809	0,018	**-3,966**	**0,000**	myosin, light polypeptide 4 [Source:MGI Symbol;Acc:MGI:97267]
ENSMUSG00000024691	Fam111a	-2,388	0,000	-2,132	0,000	-1,948	0,000	**1,044**	**0,000**	family with sequence similarity 111, member A [Source:MGI Symbol;Acc:MGI:1915508]
ENSMUSG00000041220	Elovl6	0,065	0,951	1,985	0,024	3,043	0,001	**-4,720**	**0,000**	ELOVL family member 6, elongation of long chain fatty acids (yeast) [Source:MGI Symbol;Acc:MGI:2156528]
ENSMUSG00000006403	Adamts4	-1,598	0,000	-2,273	0,000	-2,252	0,000	**1,520**	**0,000**	a disintegrin-like and metallopeptidase (reprolysin type) with thrombospondin type 1 motif, 4 [Source:MGI Symbol;Acc:MGI:1339949]
ENSMUSG00000058672	Tubb2a	-0,945	0,000	-1,542	0,000	-1,706	0,000	**1,082**	**0,000**	tubulin, beta 2A class IIA [Source:MGI Symbol;Acc:MGI:107861]
ENSMUSG00000003123	Lipe	4,438	0,000	3,780	0,000	2,375	0,000	**-1,659**	**0,000**	lipase, hormone sensitive [Source:MGI Symbol;Acc:MGI:96790]
ENSMUSG00000030963	Umod	6,316	0,000	8,499	0,000	8,305	0,000	**-2,877**	**0,000**	uromodulin [Source:MGI Symbol;Acc:MGI:102674]
ENSMUSG00000091705	H2-Q2	-7,733	0,000	-2,908	0,607	0,000	1,000	**7,609**	**0,000**	histocompatibility 2, Q region locus 2 [Source:MGI Symbol;Acc:MGI:95931]
ENSMUSG00000106749	Gm43281	2,393	0,000	2,955	0,000	2,642	0,000	**-3,661**	**0,000**	predicted gene 43281 [Source:MGI Symbol;Acc:MGI:5663418]
ENSMUSG00000073608	Gm6086	8,485	0,000	4,161	0,000	1,518	0,000	**-1,106**	**0,000**	predicted gene 6086 [Source:MGI Symbol;Acc:MGI:3646771]
ENSMUSG00000061080	Lsamp	1,343	0,000	2,062	0,000	2,983	0,000	**-1,466**	**0,000**	limbic system-associated membrane protein [Source:MGI Symbol;Acc:MGI:1261760]
ENSMUSG00000102189	Gm37194	-3,086	0,000	-3,549	0,000	-1,502	0,028	**2,931**	**0,000**	predicted gene, 37194 [Source:MGI Symbol;Acc:MGI:5610422]
ENSMUSG00000008153	Clstn3	0,826	0,404	3,705	0,001	6,321	0,000	**-5,817**	**0,000**	calsyntenin 3 [Source:MGI Symbol;Acc:MGI:2178323]
ENSMUSG00000042851	Zc3h6	1,835	0,000	1,259	0,000	0,941	0,000	**-1,047**	**0,000**	zinc finger CCCH type containing 6 [Source:MGI Symbol;Acc:MGI:1926001]
ENSMUSG00000018830	Myh11	3,061	0,000	3,999	0,000	3,392	0,000	**-1,588**	**0,000**	myosin, heavy polypeptide 11, smooth muscle [Source:MGI Symbol;Acc:MGI:102643]
ENSMUSG00000073437	D330041H03Rik	-1,576	0,000	-1,543	0,000	-0,723	0,041	**1,424**	**0,000**	RIKEN cDNA D330041H03 gene [Source:MGI Symbol;Acc:MGI:3603827]
ENSMUSG00000030208	Emp1	0,030	0,923	-1,193	0,000	-1,646	0,000	**1,204**	**0,000**	epithelial membrane protein 1 [Source:MGI Symbol;Acc:MGI:107941]
ENSMUSG00000027513	Pck1	11,008	0,000	8,328	0,000	10,053	0,000	**-4,770**	**0,000**	phosphoenolpyruvate carboxykinase 1, cytosolic [Source:MGI Symbol;Acc:MGI:97501]

ENSMUSG00000107706	RP23-62G15.1	2,397	0,000	1,489	0,002	1,466	0,004	**-2,757**	**0,000**	
ENSMUSG00000002997	Prkar2b	0,953	0,265	2,321	0,009	3,585	0,000	**-4,291**	**0,000**	protein kinase, cAMP dependent regulatory, type II beta [Source:MGI Symbol;Acc:MGI:97760]
ENSMUSG00000028132	Tmem56	-0,065	0,884	0,544	0,052	1,093	0,000	**-1,426**	**0,000**	transmembrane protein 56 [Source:MGI Symbol;Acc:MGI:1923195]
ENSMUSG00000020407	Upp1	-1,024	0,000	-2,959	0,000	-3,266	0,000	**1,210**	**0,000**	uridine phosphorylase 1 [Source:MGI Symbol;Acc:MGI:1097668]
ENSMUSG00000021702	Thbs4	-3,436	0,000	-2,867	0,000	-1,551	0,033	**3,274**	**0,000**	thrombospondin 4 [Source:MGI Symbol;Acc:MGI:1101779]
ENSMUSG00000005483	Dnajb1	-1,046	0,000	-0,948	0,000	-0,554	0,021	**1,019**	**0,000**	DnaJ (Hsp40) homolog, subfamily B, member 1 [Source:MGI Symbol;Acc:MGI:1931874]
ENSMUSG00000038393	Txnip	3,100	0,000	1,922	0,000	0,794	0,001	**-1,053**	**0,000**	thioredoxin interacting protein [Source:MGI Symbol;Acc:MGI:1889549]
ENSMUSG00000023905	Tnfrsf12a	0,826	0,002	-0,223	0,467	-0,901	0,001	**1,215**	**0,000**	tumor necrosis factor receptor superfamily, member 12a [Source:MGI Symbol;Acc:MGI:1351484]
ENSMUSG00000068745	Mybphl	-0,666	0,469	2,274	0,017	4,259	0,000	**-4,756**	**0,000**	myosin binding protein H-like [Source:MGI Symbol;Acc:MGI:1916003]
ENSMUSG00000068748	Ptprz1	-0,077	0,886	0,933	0,069	1,786	0,001	**-2,401**	**0,000**	protein tyrosine phosphatase, receptor type Z, polypeptide 1 [Source:MGI Symbol;Acc:MGI:97816]
ENSMUSG00000069378	Prdm6	-3,267	0,000	-0,391	0,441	1,709	0,014	**-4,436**	**0,000**	PR domain containing 6 [Source:MGI Symbol;Acc:MGI:2684938]
ENSMUSG00000025153	Fasn	2,455	0,006	2,981	0,001	3,009	0,001	**-4,226**	**0,000**	fatty acid synthase [Source:MGI Symbol;Acc:MGI:95485]
ENSMUSG00000104030	5330406M23Rik	5,273	0,000	4,566	0,000	5,525	0,000	**-2,035**	**0,000**	RIKEN cDNA 5330406M23 gene [Source:MGI Symbol;Acc:MGI:1923921]
ENSMUSG00000050359	Sprr1a	0,603	0,769	-2,691	0,000	0,752	0,773	**2,954**	**0,000**	small proline-rich protein 1A [Source:MGI Symbol;Acc:MGI:106660]
ENSMUSG00000001494	Sost	1,036	0,106	0,880	0,196	3,570	0,000	**-3,060**	**0,000**	sclerostin [Source:MGI Symbol;Acc:MGI:1921749]
ENSMUSG00000026827	Gpd2	1,272	0,005	1,852	0,000	1,503	0,002	**-2,056**	**0,001**	glycerol phosphate dehydrogenase 2, mitochondrial [Source:MGI Symbol;Acc:MGI:99778]
ENSMUSG00000021388	Aspn	6,680	0,000	-0,556	0,092	-1,285	0,000	**1,368**	**0,001**	asporin [Source:MGI Symbol;Acc:MGI:1913945]
ENSMUSG00000021612	Slc6a18	8,663	0,000	8,513	0,000	8,318	0,000	**-2,519**	**0,001**	solute carrier family 6 (neurotransmitter transporter), member 18 [Source:MGI Symbol;Acc:MGI:1336892]

ENSMUSG00000010492	Uckl1os	6,252	0,000	5,670	0,000	4,429	0,000	**-1,334**	**0,001**	uridine-cytidine kinase 1-like 1, opposite strand [Source:MGI Symbol;Acc:MGI:3801877]
ENSMUSG00000105962	Gm42432	3,067	0,000	2,330	0,000	2,582	0,000	**-1,577**	**0,001**	predicted gene 42432 [Source:MGI Symbol;Acc:MGI:5662569]
ENSMUSG00000020532	Acaca	1,414	0,054	2,209	0,004	2,552	0,001	**-3,389**	**0,001**	acetyl-Coenzyme A carboxylase alpha [Source:MGI Symbol;Acc:MGI:108451]
ENSMUSG00000028976	Slc2a5	0,680	0,247	1,365	0,022	0,974	0,126	**-2,827**	**0,001**	solute carrier family 2 (facilitated glucose transporter), member 5 [Source:MGI Symbol;Acc:MGI:1928369]
ENSMUSG00000000486	Sept1	-2,238	0,000	-2,751	0,000	-2,069	0,000	**1,398**	**0,001**	septin 1 [Source:MGI Symbol;Acc:MGI:1858916]
ENSMUSG00000023914	Mep1a	8,293	0,000	9,739	0,000	9,544	0,000	**-2,582**	**0,001**	meprin 1 alpha [Source:MGI Symbol;Acc:MGI:96963]
ENSMUSG00000048142	Nat8l	1,685	0,001	1,116	0,029	2,139	0,000	**-2,207**	**0,001**	N-acetyltransferase 8-like [Source:MGI Symbol;Acc:MGI:2447776]
ENSMUSG00000028194	Ddah1	-2,176	0,000	-1,479	0,000	-0,703	0,013	**1,113**	**0,001**	dimethylarginine dimethylaminohydrolase 1 [Source:MGI Symbol;Acc:MGI:1916469]
ENSMUSG00000030433	Sbk2	2,360	0,000	1,789	0,000	1,902	0,000	**-1,582**	**0,001**	SH3-binding domain kinase family, member 2 [Source:MGI Symbol;Acc:MGI:2685925]
ENSMUSG00000105445	Gm42972	1,316	0,007	-0,905	0,018	-0,421	0,337	**-2,884**	**0,001**	predicted gene 42972 [Source:MGI Symbol;Acc:MGI:5663109]
ENSMUSG00000024694	Keg1	8,701	0,000	5,022	0,000	8,357	0,000	**-2,051**	**0,001**	kidney expressed gene 1 [Source:MGI Symbol;Acc:MGI:1928492]
ENSMUSG00000064371	mt-Tt	2,565	0,000	3,367	0,000	-0,868	0,018	**-1,744**	**0,001**	mitochondrially encoded tRNA threonine [Source:MGI Symbol;Acc:MGI:102473]
ENSMUSG00000028364	Tnc	-5,761	0,000	-4,716	0,000	-4,590	0,000	**2,097**	**0,001**	tenascin C [Source:MGI Symbol;Acc:MGI:101922]
ENSMUSG00000046275	Tusc5	8,236	0,000	5,578	0,000	5,407	0,000	**-2,795**	**0,001**	tumor suppressor candidate 5 [Source:MGI Symbol;Acc:MGI:3029307]
ENSMUSG00000073485	H3f3aos	0,443	0,207	0,889	0,020	0,695	0,069	**-1,702**	**0,001**	H3 histone, family 3A, opposite strand [Source:MGI Symbol;Acc:MGI:3802006]
ENSMUSG00000038463	Olfml2b	-1,815	0,000	-1,127	0,000	-1,128	0,000	**1,193**	**0,002**	olfactomedin-like 2B [Source:MGI Symbol;Acc:MGI:2443310]
ENSMUSG00000038777	Sema6c	-0,747	0,001	0,847	0,000	0,916	0,000	**-1,005**	**0,002**	sema domain, transmembrane domain (TM), and cytoplasmic domain, (semaphorin) 6C [Source:MGI Symbol;Acc:MGI:1338032]
ENSMUSG00000102153	Gm37474	2,138	0,000	1,006	0,024	0,838	0,078	**-2,045**	**0,002**	predicted gene, 37474 [Source:MGI Symbol;Acc:MGI:5610702]

ENSMUSG00 000027966	Col11a1	-7,377	0,000	-6,280	0,000	-5,886	0,000	**3,508**	**0,002**	collagen, type XI, alpha 1 [Source:MGI Symbol;Acc:MGI:88446]
ENSMUSG00 000037071	Scd1	7,011	0,000	7,323	0,000	8,448	0,000	**-4,502**	**0,002**	stearoyl-Coenzyme A desaturase 1 [Source:MGI Symbol;Acc:MGI:98239]
ENSMUSG00 000102352	Gm3834 6	1,651	0,000	1,101	0,013	1,162	0,016	**-2,175**	**0,002**	predicted gene, 38346 [Source:MGI Symbol;Acc:MGI:5611574]
ENSMUSG00 000021957	Tkt	0,795	0,210	1,823	0,005	2,170	0,001	**-2,564**	**0,002**	transketolase [Source:MGI Symbol;Acc:MGI:105992]
ENSMUSG00 000105733	Gm4297 3	1,924	0,001	0,367	0,643	1,278	0,035	**-2,828**	**0,002**	predicted gene 42973 [Source:MGI Symbol;Acc:MGI:5663110]
ENSMUSG00 000049871	Nlrc3	0,255	0,418	-0,211	0,571	-0,795	0,007	**1,107**	**0,003**	NLR family, CARD domain containing 3 [Source:MGI Symbol;Acc:MGI:2444070]
ENSMUSG00 000030935	Acsm3	8,228	0,000	4,886	0,000	9,478	0,000	**-2,902**	**0,003**	acyl-CoA synthetase medium-chain family member 3 [Source:MGI Symbol;Acc:MGI:99538]
ENSMUSG00 000026043	Col3a1	-1,031	0,001	-1,715	0,000	-1,926	0,000	**1,197**	**0,003**	collagen, type III, alpha 1 [Source:MGI Symbol;Acc:MGI:88453]
ENSMUSG00 000106826	Gm4258 3	1,937	0,000	1,238	0,017	2,073	0,000	**-2,366**	**0,003**	predicted gene 42583 [Source:MGI Symbol;Acc:MGI:5662720]
ENSMUSG00 000037166	Ppp1r14 a	1,209	0,000	1,105	0,000	0,854	0,005	**-1,185**	**0,003**	protein phosphatase 1, regulatory (inhibitor) subunit 14A [Source:MGI Symbol;Acc:MGI:1931139]
ENSMUSG00 000059824	Dbp	3,905	0,000	2,842	0,000	2,094	0,000	**-1,355**	**0,003**	D site albumin promoter binding protein [Source:MGI Symbol;Acc:MGI:94866]
ENSMUSG00 000052520	Cyp2j5	7,264	0,000	7,057	0,000	8,506	0,000	**-1,743**	**0,003**	cytochrome P450, family 2, subfamily j, polypeptide 5 [Source:MGI Symbol;Acc:MGI:1270149]
ENSMUSG00 000022613	Miox	7,776	0,000	5,233	0,000	7,433	0,000	**-1,628**	**0,003**	myo-inositol oxygenase [Source:MGI Symbol;Acc:MGI:1891725]
ENSMUSG00 000025900	Rp1	3,332	0,000	5,185	0,000	4,016	0,000	**-1,725**	**0,004**	retinitis pigmentosa 1 (human) [Source:MGI Symbol;Acc:MGI:1341105]
ENSMUSG00 000072949	Acot1	0,755	0,030	0,269	0,484	-1,531	0,000	**-1,411**	**0,004**	acyl-CoA thioesterase 1 [Source:MGI Symbol;Acc:MGI:1349396]
ENSMUSG00 000032085	Tagln	-0,985	0,003	1,478	0,000	1,634	0,000	**-1,303**	**0,004**	transgelin [Source:MGI Symbol;Acc:MGI:106012]
ENSMUSG00 000032758	Kap	12,243	0,000	12,093	0,000	11,898	0,000	**-1,991**	**0,004**	kidney androgen regulated protein [Source:MGI Symbol;Acc:MGI:96653]
ENSMUSG00 000027171	Prrg4	0,902	0,009	0,675	0,060	-0,446	0,243	**-1,530**	**0,004**	proline rich Gla (G-carboxyglutamic acid) 4 (transmembrane) [Source:MGI Symbol;Acc:MGI:2442211]

ENSMUSG00000105119	Gm43765	3,156	0,000	1,343	0,023	0,643	0,297	**-3,346**	**0,004**	predicted gene 43765 [Source:MGI Symbol;Acc:MGI:5663902]
ENSMUSG00000086596	Susd5	4,190	0,000	5,364	0,000	6,461	0,000	**-2,165**	**0,004**	sushi domain containing 5 [Source:MGI Symbol;Acc:MGI:2685972]
ENSMUSG00000039323	Igfbp2	-5,416	0,000	-1,863	0,000	1,586	0,016	**-2,901**	**0,004**	insulin-like growth factor binding protein 2 [Source:MGI Symbol;Acc:MGI:96437]
ENSMUSG00000023019	Gpd1	7,624	0,000	6,154	0,000	4,208	0,000	**-2,245**	**0,004**	glycerol-3-phosphate dehydrogenase 1 (soluble) [Source:MGI Symbol;Acc:MGI:95679]
ENSMUSG00000056174	Col8a2	-3,325	0,000	-1,139	0,117	-0,087	0,993	**2,584**	**0,004**	collagen, type VIII, alpha 2 [Source:MGI Symbol;Acc:MGI:88464]
ENSMUSG00000107742	RP23-89F9.2	0,744	0,026	-0,093	0,882	-0,648	0,049	**-1,479**	**0,005**	
ENSMUSG00000104271	Gm37891	0,932	0,013	0,089	0,859	-0,546	0,129	**-1,705**	**0,005**	predicted gene, 37891 [Source:MGI Symbol;Acc:MGI:5611119]
ENSMUSG00000106121	Gm42679	3,598	0,000	1,694	0,001	1,145	0,029	**-2,269**	**0,005**	predicted gene 42679 [Source:MGI Symbol;Acc:MGI:5662816]
ENSMUSG00000097284	4930480K23Rik	1,117	0,000	-0,056	0,825	-0,300	0,264	**-1,127**	**0,005**	RIKEN cDNA 4930480K23 gene [Source:MGI Symbol;Acc:MGI:1922266]
ENSMUSG00000033208	S100b	4,384	0,000	3,443	0,000	3,254	0,000	**-2,802**	**0,005**	S100 protein, beta polypeptide, neural [Source:MGI Symbol;Acc:MGI:98217]
ENSMUSG00000022821	Hgd	2,387	0,000	0,266	0,525	3,339	0,000	**-2,093**	**0,005**	homogentisate 1, 2-dioxygenase [Source:MGI Symbol;Acc:MGI:96078]
ENSMUSG00000018740	Slc25a35	3,589	0,000	3,064	0,000	3,101	0,000	**-1,038**	**0,005**	solute carrier family 25, member 35 [Source:MGI Symbol;Acc:MGI:1919248]
ENSMUSG00000103593	Gm37352	2,718	0,000	1,550	0,000	1,105	0,000	**-1,020**	**0,005**	predicted gene, 37352 [Source:MGI Symbol;Acc:MGI:5610580]
ENSMUSG00000044471	Lincpint	4,743	0,000	6,921	0,000	4,228	0,000	**-2,119**	**0,005**	long intergenic non-protein coding RNA, Trp53 induced transcript [Source:MGI Symbol;Acc:MGI:2673128]
ENSMUSG00000049336	Tenm2	-3,668	0,000	-1,233	0,002	1,582	0,015	**-2,662**	**0,005**	teneurin transmembrane protein 2 [Source:MGI Symbol;Acc:MGI:1345184]
ENSMUSG00000036083	Slc17a3	7,614	0,000	2,534	0,000	7,271	0,000	**-1,693**	**0,005**	solute carrier family 17 (sodium phosphate), member 3 [Source:MGI Symbol;Acc:MGI:2389216]
ENSMUSG00000024650	Slc22a6	7,491	0,000	7,341	0,000	7,148	0,000	**-1,644**	**0,006**	solute carrier family 22 (organic anion transporter), member 6 [Source:MGI Symbol;Acc:MGI:892001]
ENSMUSG00000029335	Bmp3	-0,648	0,406	1,519	0,075	3,641	0,001	**-3,911**	**0,006**	bone morphogenetic protein 3 [Source:MGI Symbol;Acc:MGI:88179]

ENSMUSG00000042436	Mfap4	-1,770	0,001	-0,749	0,194	-0,295	0,659	**2,018**	**0,006**	microfibrillar-associated protein 4 [Source:MGI Symbol;Acc:MGI:1342276]
ENSMUSG00000020917	Acly	0,704	0,257	1,489	0,018	1,703	0,008	**-2,298**	**0,006**	ATP citrate lyase [Source:MGI Symbol;Acc:MGI:103251]
ENSMUSG00000085558	4930412C18Rik	1,771	0,000	1,402	0,000	1,662	0,000	**-1,514**	**0,006**	RIKEN cDNA 4930412C18 gene [Source:MGI Symbol;Acc:MGI:2443490]
ENSMUSG00000060224	Pyroxd2	-0,626	0,101	-1,746	0,000	-0,968	0,013	**1,355**	**0,006**	pyridine nucleotide-disulphide oxidoreductase domain 2 [Source:MGI Symbol;Acc:MGI:1921830]
ENSMUSG00000033022	Cdo1	0,973	0,251	3,273	0,000	3,985	0,000	**-3,245**	**0,006**	cysteine dioxygenase 1, cytosolic [Source:MGI Symbol;Acc:MGI:105925]
ENSMUSG00000054675	Tmem119	-2,716	0,000	-0,951	0,074	-0,974	0,077	**1,858**	**0,007**	transmembrane protein 119 [Source:MGI Symbol;Acc:MGI:2385228]
ENSMUSG00000024131	Slc3a1	2,618	0,000	2,547	0,000	4,001	0,000	**-2,500**	**0,007**	solute carrier family 3, member 1 [Source:MGI Symbol;Acc:MGI:1195264]
ENSMUSG00000029838	Ptn	-5,606	0,000	-4,119	0,000	-3,750	0,000	**1,938**	**0,007**	pleiotrophin [Source:MGI Symbol;Acc:MGI:97804]
ENSMUSG00000047976	Kcna1	1,821	0,000	6,581	0,000	4,665	0,000	**-1,482**	**0,008**	potassium voltage-gated channel, shaker-related subfamily, member 1 [Source:MGI Symbol;Acc:MGI:96654]
ENSMUSG00000026042	Col5a2	-2,293	0,000	-1,984	0,000	-1,997	0,000	**1,092**	**0,008**	collagen, type V, alpha 2 [Source:MGI Symbol;Acc:MGI:88458]
ENSMUSG00000067199	Frat1	0,465	0,108	1,284	0,000	0,989	0,003	**-1,181**	**0,008**	frequently rearranged in advanced T cell lymphomas [Source:MGI Symbol;Acc:MGI:109450]
ENSMUSG00000026390	Marco	3,200	0,335	-3,348	0,010	-0,093	1,000	**4,226**	**0,008**	macrophage receptor with collagenous structure [Source:MGI Symbol;Acc:MGI:1309998]
ENSMUSG00000001131	Timp1	-2,093	0,000	-1,470	0,011	-2,078	0,000	**2,035**	**0,008**	tissue inhibitor of metalloproteinase 1 [Source:MGI Symbol;Acc:MGI:98752]
ENSMUSG00000028179	Cth	-0,009	1,000	1,751	0,000	5,385	0,000	**-1,570**	**0,008**	cystathionase (cystathionine gamma-lyase) [Source:MGI Symbol;Acc:MGI:1339968]
ENSMUSG00000007888	Crlf1	-2,666	0,000	1,915	0,039	3,239	0,002	**2,466**	**0,008**	cytokine receptor-like factor 1 [Source:MGI Symbol;Acc:MGI:1340030]
ENSMUSG00000002204	Napsa	6,943	0,000	5,147	0,000	4,879	0,000	**-1,968**	**0,009**	napsin A aspartic peptidase [Source:MGI Symbol;Acc:MGI:109365]
ENSMUSG00000022419	Deptor	3,645	0,000	3,164	0,000	3,055	0,000	**-1,273**	**0,009**	DEP domain containing MTOR-interacting protein [Source:MGI Symbol;Acc:MGI:2146322]
ENSMUSG00000051048	P4ha3	-6,530	0,000	-6,614	0,000	-5,451	0,000	**2,616**	**0,009**	procollagen-proline, 2-oxoglutarate 4-dioxygenase (proline 4-hydroxylase), alpha polypeptide III [Source:MGI Symbol;Acc:MGI:2444049]

ENSMUSG00000027996	Sfrp2	-2,940	0,000	-2,008	0,002	-0,891	0,194	**2,223**	**0,009**	secreted frizzled-related protein 2 [Source:MGI Symbol;Acc:MGI:108078]
ENSMUSG00000037493	Cib2	1,069	0,152	2,716	0,001	3,032	0,000	**-2,788**	**0,010**	calcium and integrin binding family member 2 [Source:MGI Symbol;Acc:MGI:1929293]
ENSMUSG00000097467	Gm26737	-3,829	0,000	-4,059	0,000	-3,516	0,000	**3,001**	**0,010**	predicted gene, 26737 [Source:MGI Symbol;Acc:MGI:5477231]
ENSMUSG00000038009	Dnajc22	-0,369	0,355	0,645	0,367	2,595	0,001	**-3,317**	**0,010**	DnaJ (Hsp40) homolog, subfamily C, member 22 [Source:MGI Symbol;Acc:MGI:1920028]
ENSMUSG00000045294	Insig1	0,485	0,099	0,785	0,009	0,980	0,001	**-1,031**	**0,010**	insulin induced gene 1 [Source:MGI Symbol;Acc:MGI:1916289]
ENSMUSG00000024526	Cidea	8,013	0,000	3,467	0,000	1,605	0,001	**-1,582**	**0,010**	cell death-inducing DNA fragmentation factor, alpha subunit-like effector A [Source:MGI Symbol;Acc:MGI:1270845]
ENSMUSG00000029123	Stk32b	-0,175	0,576	1,039	0,006	1,911	0,000	**-1,430**	**0,011**	serine/threonine kinase 32B [Source:MGI Symbol;Acc:MGI:1927552]
ENSMUSG00000028434	Epb41l4b	0,521	0,045	0,923	0,001	-0,583	0,016	**-1,016**	**0,011**	erythrocyte membrane protein band 4.1 like 4b [Source:MGI Symbol;Acc:MGI:1859149]
ENSMUSG00000105597	4633401B06Rik	2,617	0,000	1,661	0,000	0,330	0,314	**-1,105**	**0,011**	RIKEN cDNA 4633401B06 gene [Source:MGI Symbol;Acc:MGI:1918078]
ENSMUSG00000045968	Teddm2	3,698	0,000	3,083	0,000	2,209	0,000	**-1,646**	**0,011**	transmembrane epididymal family member 2 [Source:MGI Symbol;Acc:MGI:1923273]
ENSMUSG00000003528	Slc25a1	1,372	0,035	2,393	0,000	2,951	0,000	**-2,307**	**0,012**	solute carrier family 25 (mitochondrial carrier, citrate transporter), member 1 [Source:MGI Symbol;Acc:MGI:1345283]
ENSMUSG00000038677	Scube3	-2,764	0,000	1,948	0,001	2,999	0,000	**-2,041**	**0,013**	signal peptide, CUB domain, EGF-like 3 [Source:MGI Symbol;Acc:MGI:3045253]
ENSMUSG00000040690	Col16a1	-1,879	0,000	-1,502	0,000	-1,092	0,001	**1,100**	**0,013**	collagen, type XVI, alpha 1 [Source:MGI Symbol;Acc:MGI:1095396]
ENSMUSG00000042429	Adora1	1,062	0,000	-0,673	0,028	0,249	0,493	**-1,049**	**0,014**	adenosine A1 receptor [Source:MGI Symbol;Acc:MGI:99401]
ENSMUSG00000024747	Aldh1a7	1,299	0,257	3,883	0,003	4,310	0,001	**-4,478**	**0,014**	aldehyde dehydrogenase family 1, subfamily A7 [Source:MGI Symbol;Acc:MGI:1347050]
ENSMUSG00000074604	Mgst2	1,183	0,048	-0,598	0,188	1,982	0,014	**-2,745**	**0,015**	microsomal glutathione S-transferase 2 [Source:MGI Symbol;Acc:MGI:2448481]
ENSMUSG00000078161	Erich3	1,447	0,001	0,896	0,039	1,597	0,001	**-1,619**	**0,015**	glutamate rich 3 [Source:MGI Symbol;Acc:MGI:1919095]
ENSMUSG00000024892	Pcx	1,577	0,000	1,858	0,000	3,590	0,000	**-1,085**	**0,015**	pyruvate carboxylase [Source:MGI Symbol;Acc:MGI:97520]

ENSMUSG00000037625	Cldn11	-5,961	0,000	-4,970	0,000	-4,231	0,000	**3,689**	**0,016**	claudin 11 [Source:MGI Symbol;Acc:MGI:106925]
ENSMUSG00000026773	Pfkfb3	0,325	0,488	0,715	0,129	2,028	0,000	**-1,508**	**0,016**	6-phosphofructo-2-kinase/fructose-2,6-biphosphatase 3 [Source:MGI Symbol;Acc:MGI:2181202]
ENSMUSG00000026069	Il1rl1	2,244	0,000	0,856	0,018	1,832	0,000	**-1,296**	**0,016**	interleukin 1 receptor-like 1 [Source:MGI Symbol;Acc:MGI:98427]
ENSMUSG00000027750	Postn	-0,996	0,049	-3,286	0,000	-3,515	0,000	**1,703**	**0,016**	periostin, osteoblast specific factor [Source:MGI Symbol;Acc:MGI:1926321]
ENSMUSG00000020774	Aspa	6,198	0,000	5,977	0,000	7,296	0,000	**-1,295**	**0,017**	aspartoacylase [Source:MGI Symbol;Acc:MGI:87914]
ENSMUSG00000030945	Acsm2	10,181	0,000	10,031	0,000	9,836	0,000	**-1,958**	**0,017**	acyl-CoA synthetase medium-chain family member 2 [Source:MGI Symbol;Acc:MGI:2385289]
ENSMUSG00000108365	RP24-174G2.2	1,580	0,000	1,516	0,001	1,267	0,006	**-1,561**	**0,018**	
ENSMUSG00000103674	Gm37699	2,465	0,000	0,610	0,181	0,257	0,686	**-1,654**	**0,018**	predicted gene, 37699 [Source:MGI Symbol;Acc:MGI:5610927]
ENSMUSG00000044349	Snhg11	-0,502	0,392	4,810	0,000	2,150	0,001	**-1,994**	**0,019**	small nucleolar RNA host gene 11 [Source:MGI Symbol;Acc:MGI:2441845]
ENSMUSG00000040724	Kcna2	3,239	0,000	4,560	0,000	5,281	0,000	**-1,355**	**0,020**	potassium voltage-gated channel, shaker-related subfamily, member 2 [Source:MGI Symbol;Acc:MGI:96659]
ENSMUSG00000017950	Hnf4a	0,845	0,125	-0,391	0,523	7,312	0,000	**-1,893**	**0,020**	hepatic nuclear factor 4, alpha [Source:MGI Symbol;Acc:MGI:109128]
ENSMUSG00000032942	Ucp3	10,354	0,000	8,668	0,000	1,555	0,000	**-1,290**	**0,020**	uncoupling protein 3 (mitochondrial, proton carrier) [Source:MGI Symbol;Acc:MGI:1099787]
ENSMUSG00000019303	Psmc3ip	-1,969	0,000	-1,362	0,001	-1,277	0,003	**1,345**	**0,020**	proteasome (prosome, macropain) 26S subunit, ATPase 3, interacting protein [Source:MGI Symbol;Acc:MGI:1098610]
ENSMUSG00000025194	Abcc2	1,949	0,001	1,088	0,082	7,915	0,000	**-2,110**	**0,020**	ATP-binding cassette, sub-family C (CFTR/MRP), member 2 [Source:MGI Symbol;Acc:MGI:1352447]
ENSMUSG00000063594	Gng8	-2,003	0,000	-0,828	0,178	0,048	1,000	**1,734**	**0,020**	guanine nucleotide binding protein (G protein), gamma 8 [Source:MGI Symbol;Acc:MGI:109163]
ENSMUSG00000106490	Gm43283	1,463	0,003	0,837	0,110	0,839	0,157	**-1,966**	**0,021**	predicted gene 43283 [Source:MGI Symbol;Acc:MGI:5663420]
ENSMUSG00000102516	Gm38340	-0,880	0,017	1,497	0,001	1,182	0,009	**-1,469**	**0,021**	predicted gene, 38340 [Source:MGI Symbol;Acc:MGI:5611568]
ENSMUSG00000097993	Ptprv	-1,366	0,014	0,008	1,000	0,406	0,733	**1,840**	**0,021**	protein tyrosine phosphatase, receptor type, V [Source:MGI Symbol;Acc:MGI:108027]

ENSMUSG00000006216	Clcnkb	2,898	0,000	0,617	0,067	0,095	0,771	**-1,169**	**0,021**	chloride channel, voltage-sensitive Kb [Source:MGI Symbol;Acc:MGI:1930643]
ENSMUSG00000020155	Kcnmb1	-1,210	0,000	1,217	0,002	1,298	0,002	**-1,303**	**0,021**	potassium large conductance calcium-activated channel, subfamily M, beta member 1 [Source:MGI Symbol;Acc:MGI:1334203]
ENSMUSG00000103123	Gm37390	2,739	0,000	1,128	0,042	1,575	0,010	**-2,044**	**0,021**	predicted gene, 37390 [Source:MGI Symbol;Acc:MGI:5610618]
ENSMUSG00000007682	Dio2	-0,229	0,713	-1,912	0,001	-0,915	0,121	**1,758**	**0,022**	deiodinase, iodothyronine, type II [Source:MGI Symbol;Acc:MGI:1338833]
ENSMUSG00000038298	Pdzk1	4,071	0,000	1,896	0,001	4,447	0,000	**-1,911**	**0,022**	PDZ domain containing 1 [Source:MGI Symbol;Acc:MGI:1928901]
ENSMUSG00000088689	Scarna17	2,547	0,000	1,428	0,003	1,183	0,025	**-1,852**	**0,022**	small Cajal body-specific RNA 17 [Source:MGI Symbol;Acc:MGI:3819483]
ENSMUSG00000050914	Ankrd37	-0,072	0,954	-0,997	0,001	-0,642	0,062	**1,010**	**0,022**	ankyrin repeat domain 37 [Source:MGI Symbol;Acc:MGI:3603344]
ENSMUSG00000005045	Chd5	0,726	0,062	1,992	0,000	0,692	0,119	**-1,520**	**0,022**	chromodomain helicase DNA binding protein 5 [Source:MGI Symbol;Acc:MGI:3036258]
ENSMUSG00000041653	Pnpla3	4,638	0,000	6,200	0,000	7,178	0,000	**-2,515**	**0,022**	patatin-like phospholipase domain containing 3 [Source:MGI Symbol;Acc:MGI:2151796]
ENSMUSG00000028047	Thbs3	-1,290	0,000	-1,240	0,000	-1,609	0,000	**1,025**	**0,022**	thrombospondin 3 [Source:MGI Symbol;Acc:MGI:98739]
ENSMUSG00000043621	Ubxn10	1,033	0,002	1,112	0,002	1,576	0,000	**-1,185**	**0,022**	UBX domain protein 10 [Source:MGI Symbol;Acc:MGI:2443123]
ENSMUSG00000001420	Tmem79	1,670	0,012	2,597	0,000	2,991	0,000	**-2,279**	**0,024**	transmembrane protein 79 [Source:MGI Symbol;Acc:MGI:1919163]
ENSMUSG00000103772	Gm36933	1,796	0,000	0,566	0,270	1,599	0,001	**-1,605**	**0,025**	predicted gene, 36933 [Source:MGI Symbol;Acc:MGI:5610161]
ENSMUSG00000031766	Slc12a3	3,542	0,000	3,304	0,000	3,845	0,000	**-1,869**	**0,026**	solute carrier family 12, member 3 [Source:MGI Symbol;Acc:MGI:108114]
ENSMUSG00000033356	Pus7l	-1,769	0,000	-1,089	0,001	-1,222	0,000	**1,039**	**0,026**	pseudouridylate synthase 7 homolog (S. cerevisiae)-like [Source:MGI Symbol;Acc:MGI:1926145]
ENSMUSG00000037686	Aspg	2,649	0,009	5,584	0,000	5,979	0,000	**-3,408**	**0,026**	asparaginase homolog (S. cerevisiae) [Source:MGI Symbol;Acc:MGI:2144822]
ENSMUSG00000037353	Letmd1	0,807	0,099	1,404	0,005	1,535	0,003	**-1,549**	**0,027**	LETM1 domain containing 1 [Source:MGI Symbol;Acc:MGI:1915864]
ENSMUSG00000028957	Per3	1,659	0,000	0,801	0,014	0,229	0,549	**-1,003**	**0,027**	period circadian clock 3 [Source:MGI Symbol;Acc:MGI:1277134]

ENSMUSG00000017453	Pipox	1,066	0,003	-0,419	0,325	0,448	0,248	**-1,266**	**0,027**	pipecolic acid oxidase [Source:MGI Symbol;Acc:MGI:1197006]
ENSMUSG00000032549	Rab6b	1,585	0,007	1,784	0,003	1,882	0,002	**-1,873**	**0,028**	RAB6B, member RAS oncogene family [Source:MGI Symbol;Acc:MGI:107283]
ENSMUSG00000087382	Ctcflos	1,457	0,050	0,189	0,850	2,089	0,009	**-2,457**	**0,028**	CCCTC-binding factor (zinc finger protein)-like, opposite strand [Source:MGI Symbol;Acc:MGI:1921411]
ENSMUSG00000057715	A830018L16Rik	0,071	1,000	1,869	0,011	2,483	0,005	**-3,203**	**0,029**	RIKEN cDNA A830018L16 gene [Source:MGI Symbol;Acc:MGI:2444149]
ENSMUSG00000106706	C530043K16Rik	1,856	0,000	1,620	0,002	0,887	0,076	**-1,639**	**0,029**	RIKEN cDNA C530043K16 gene [Source:MGI Symbol;Acc:MGI:2444587]
ENSMUSG00000107624	RP23-170C11.3	6,222	0,000	2,626	0,000	2,244	0,000	**-1,191**	**0,029**	
ENSMUSG00000040016	Ptger3	1,946	0,045	5,207	0,000	5,421	0,000	**-3,183**	**0,029**	prostaglandin E receptor 3 (subtype EP3) [Source:MGI Symbol;Acc:MGI:97795]
ENSMUSG00000026922	Agpat2	3,766	0,000	3,153	0,000	2,783	0,000	**-1,115**	**0,029**	1-acylglycerol-3-phosphate O-acyltransferase 2 (lysophosphatidic acid acyltransferase, beta) [Source:MGI Symbol;Acc:MGI:1914762]
ENSMUSG00000053469	Tg	-0,744	0,027	-0,903	0,008	-1,437	0,000	**1,046**	**0,030**	thyroglobulin [Source:MGI Symbol;Acc:MGI:98733]
ENSMUSG00000005124	Wisp1	-5,145	0,000	-5,050	0,000	-4,974	0,000	**1,145**	**0,031**	WNT1 inducible signaling pathway protein 1 [Source:MGI Symbol;Acc:MGI:1197008]
ENSMUSG00000044566	Cage1	-0,750	0,104	-0,896	0,068	-0,972	0,045	**1,390**	**0,031**	cancer antigen 1 [Source:MGI Symbol;Acc:MGI:1918463]
ENSMUSG00000073460	Pnldc1	0,636	0,231	1,321	0,017	0,820	0,161	**-1,755**	**0,033**	poly(A)-specific ribonuclease (PARN)-like domain containing 1 [Source:MGI Symbol;Acc:MGI:2685159]
ENSMUSG00000059049	Frem1	-6,509	0,000	-5,095	0,000	-4,595	0,000	**1,690**	**0,033**	Fras1 related extracellular matrix protein 1 [Source:MGI Symbol;Acc:MGI:2670972]
ENSMUSG00000005057	Sh2b2	0,679	0,364	2,694	0,001	2,441	0,002	**-2,342**	**0,034**	SH2B adaptor protein 2 [Source:MGI Symbol;Acc:MGI:1345171]
ENSMUSG00000101257	2310015K22Rik	6,326	0,000	3,781	0,000	4,248	0,000	**-2,173**	**0,034**	RIKEN cDNA 2310015K22 gene [Source:MGI Symbol;Acc:MGI:1916815]
ENSMUSG00000031725	Ces1f	4,427	0,000	8,828	0,000	8,633	0,000	**-2,554**	**0,034**	carboxylesterase 1F [Source:MGI Symbol;Acc:MGI:2142687]
ENSMUSG00000003279	Dlgap1	1,516	0,005	3,797	0,000	3,507	0,000	**-1,833**	**0,035**	discs, large (Drosophila) homolog-associated protein 1 [Source:MGI Symbol;Acc:MGI:1346065]
ENSMUSG00000021676	Iqgap2	-3,004	0,000	-0,627	0,157	0,396	0,431	**1,264**	**0,035**	IQ motif containing GTPase activating protein 2 [Source:MGI Symbol;Acc:MGI:2449975]

ENSMUSG00 000030688	Stard10	0,316	0,363	0,836	0,015	1,520	0,000	**-1,030**	**0,035**	START domain containing 10 [Source:MGI Symbol;Acc:MGI:1860093]
ENSMUSG00 000015970	Chdh	5,188	0,000	1,731	0,045	4,660	0,000	**-2,653**	**0,035**	choline dehydrogenase [Source:MGI Symbol;Acc:MGI:1860776]
ENSMUSG00 000105199	Gm4358 1	1,342	0,009	0,957	0,081	-1,069	0,012	**-1,798**	**0,035**	predicted gene 43581 [Source:MGI Symbol;Acc:MGI:5663718]
ENSMUSG00 000102091	Olfr1034	4,444	0,000	2,859	0,000	3,614	0,000	**-1,290**	**0,038**	olfactory receptor 1034 [Source:MGI Symbol;Acc:MGI:3030868]
ENSMUSG00 000026834	Acvr1c	-0,457	0,632	2,362	0,019	2,697	0,010	**-3,048**	**0,039**	activin A receptor, type IC [Source:MGI Symbol;Acc:MGI:2661081]
ENSMUSG00 000031938	4931406 C07Rik	0,877	0,019	1,129	0,003	0,788	0,050	**-1,142**	**0,039**	RIKEN cDNA 4931406C07 gene [Source:MGI Symbol;Acc:MGI:1918234]
ENSMUSG00 000103151	Gm3829 2	0,874	0,132	0,747	0,264	1,688	0,011	**-2,001**	**0,040**	predicted gene, 38292 [Source:MGI Symbol;Acc:MGI:5611520]
ENSMUSG00 000093672	Gm2065 5	1,636	0,000	-0,033	1,000	-0,066	1,000	**-1,535**	**0,040**	predicted gene 20655 [Source:MGI Symbol;Acc:MGI:5313102]
ENSMUSG00 000043993	2900052 L18Rik	0,314	0,318	0,655	0,039	-0,598	0,038	**-1,037**	**0,040**	RIKEN cDNA 2900052L18 gene [Source:MGI Symbol;Acc:MGI:1924085]
ENSMUSG00 000031548	Sfrp1	-3,096	0,000	-2,102	0,000	-1,228	0,001	**1,063**	**0,040**	secreted frizzled-related protein 1 [Source:MGI Symbol;Acc:MGI:892014]
ENSMUSG00 000104012	Gm3736 4	1,997	0,005	-1,365	0,002	-1,352	0,004	**-2,428**	**0,042**	predicted gene, 37364 [Source:MGI Symbol;Acc:MGI:5610592]
ENSMUSG00 000026527	Rgs7	2,509	0,000	3,623	0,000	3,070	0,000	**-1,618**	**0,042**	regulator of G protein signaling 7 [Source:MGI Symbol;Acc:MGI:1346089]
ENSMUSG00 000057963	Itpk1	1,390	0,000	1,244	0,000	1,975	0,000	**-1,021**	**0,042**	inositol 1,3,4-triphosphate 5/6 kinase [Source:MGI Symbol;Acc:MGI:2446159]
ENSMUSG00 000026582	Sele	4,643	0,000	-0,885	0,002	-1,321	0,000	**-1,019**	**0,042**	selectin, endothelial cell [Source:MGI Symbol;Acc:MGI:98278]
ENSMUSG00 000032332	Col12a1	-4,464	0,000	-3,710	0,000	-2,538	0,000	**1,919**	**0,043**	collagen, type XII, alpha 1 [Source:MGI Symbol;Acc:MGI:88448]
ENSMUSG00 000029223	Uchl1	-2,710	0,000	-1,998	0,000	-1,227	0,001	**1,039**	**0,043**	ubiquitin carboxy-terminal hydrolase L1 [Source:MGI Symbol;Acc:MGI:103149]
ENSMUSG00 000091890	A830073 O21Rik	2,076	0,000	3,870	0,000	1,357	0,001	**-1,197**	**0,046**	RIKEN cDNA A830073O21 gene [Source:MGI Symbol;Acc:MGI:2443692]
ENSMUSG00 000039476	Prrx2	-6,961	0,000	-4,586	0,000	-2,755	0,000	**2,043**	**0,046**	paired related homeobox 2 [Source:MGI Symbol;Acc:MGI:98218]

ENSMUSG00000058656	Samd12	2,896	0,000	3,051	0,000	6,647	0,000	**-1,533**	**0,048**	sterile alpha motif domain containing 12 [Source:MGI Symbol;Acc:MGI:2444518]
ENSMUSG00000040276	Pacsin1	-1,950	0,000	1,270	0,177	1,349	0,163	**1,492**	**0,048**	protein kinase C and casein kinase substrate in neurons 1 [Source:MGI Symbol;Acc:MGI:1345181]
ENSMUSG00000053930	Shisa6	-0,755	0,064	3,237	0,000	3,323	0,000	**-1,306**	**0,048**	shisa family member 6 [Source:MGI Symbol;Acc:MGI:2685725]
ENSMUSG00000020911	Krt19	-3,940	0,000	-2,363	0,000	0,056	1,000	**1,820**	**0,049**	keratin 19 [Source:MGI Symbol;Acc:MGI:96693]
ENSMUSG00000073402	Gm8909	-2,928	0,030	1,924	0,554	3,393	0,413	**3,964**	**0,049**	predicted gene 8909 [Source:MGI Symbol;Acc:MGI:3704134]
ENSMUSG00000026839	Upp2	0,587	0,097	0,202	0,674	0,907	0,026	**-1,142**	**0,049**	uridine phosphorylase 2 [Source:MGI Symbol;Acc:MGI:1923904]
ENSMUSG00000097877	Gm26703	3,307	0,000	1,543	0,000	0,759	0,083	**-1,233**	**0,049**	predicted gene, 26703 [Source:MGI Symbol;Acc:MGI:5477197]
ENSMUSG00000078190	Dnm3os	-2,988	0,000	-0,707	0,052	-1,454	0,000	**1,007**	**0,050**	dynamin 3, opposite strand [Source:MGI Symbol;Acc:MGI:3052332]
ENSMUSG00000041737	Tmem45b	10,500	0,000	10,349	0,000	10,155	0,000	**-3,774**	**0,050**	transmembrane protein 45b [Source:MGI Symbol;Acc:MGI:2384574]
ENSMUSG00000034317	Trim59	-5,501	0,000	-4,752	0,000	-3,833	0,000	**1,120**	**0,050**	tripartite motif-containing 59 [Source:MGI Symbol;Acc:MGI:1914199]
ENSMUSG00000001506	Col1a1	-1,884	0,000	-2,516	0,000	-1,796	0,000	**1,043**	**0,050**	collagen, type I, alpha 1 [Source:MGI Symbol;Acc:MGI:88467]
